# The energetics and ion coupling of cholesterol transport through Patched1

**DOI:** 10.1126/sciadv.adh1609

**Published:** 2023-08-23

**Authors:** T. Bertie Ansell, Robin A. Corey, Lucrezia Vittoria Viti, Maia Kinnebrew, Rajat Rohatgi, Christian Siebold, Mark S. P. Sansom

**Affiliations:** ^1^ Department of Biochemistry, University of Oxford, South Parks Road, Oxford OX1 3QU, UK.; ^2^ School of Physiology, Pharmacology and Neuroscience, Bristol University, Bristol BS8 1TD, UK.; ^3^ Division of Structural Biology, Wellcome Centre for Human Genetics, Roosevelt Drive, Oxford OX3 7BN, UK.; ^4^ Departments of Biochemistry and Medicine, Stanford University School of Medicine, Stanford, CA, USA.

## Abstract

Patched1 (PTCH1) is a tumor suppressor protein of the mammalian Hedgehog (HH) signaling pathway, implicated in embryogenesis and tissue homeostasis. PTCH1 inhibits the G protein–coupled receptor Smoothened (SMO) via a debated mechanism involving modulating ciliary cholesterol accessibility. Using extensive molecular dynamics simulations and free energy calculations to evaluate cholesterol transport through PTCH1, we find an energetic barrier of ~15 to 20 kilojoule per mole for cholesterol export. In silico data are coupled to in vivo biochemical assays of PTCH1 mutants to probe coupling between cation binding sites, transmembrane motions, and PTCH1 activity. Using complementary simulations of Dispatched1, we find that transition between “inward-open” and solvent “occluded” states is accompanied by Na^+^-induced pinching of intracellular helical segments. Thus, our findings illuminate the energetics and ion coupling stoichiometries of PTCH1 transport mechanisms, whereby one to three Na^+^ or two to three K^+^ couple to cholesterol export, and provide the first molecular description of transitions between distinct transport states.

## Teaser

Simulations reveal a mechanism of cholesterol transport through Patched1.

## INTRODUCTION

**Fig. 1.**
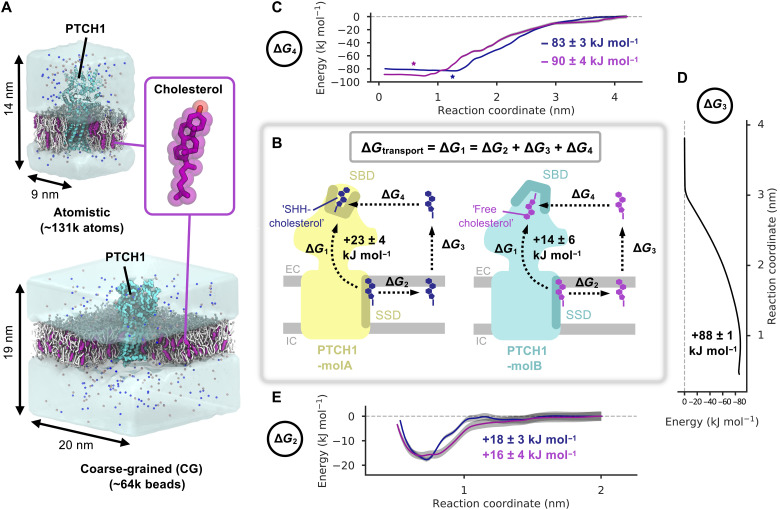
PTCH1 and the energetics of cholesterol transport—the indirect pathway. (**A**) Coarse-grained (CG) and atomistic simulation setups of PTCH1 [light blue, PDB: 6RVD (CG) (*15*)/6DMY (atomistic) (*5*)] embedded in 3:1 POPC:cholesterol (white/purple) bilayers. Water is shown as the transparent surface, and Na^+^/Cl^−^ ions are shown as blue/salmon spheres, respectively. The inset shows the structure of cholesterol. (**B**) Schematic diagram of the free energy changes associated with cholesterol movement between the PTCH1 sterol-sensing domain (SSD) and the sterol-binding domain (SBD) for the direct (Δ*G*_1_) and indirect (Δ*G*_2_, Δ*G*_3_, and Δ*G*_4_) pathways. PTCH1-molA (yellow) and PTCH1-molB (light blue) are shown with either the SHH-cholesterol (dark blue) or the free cholesterol (purple) molecules positioned in the SBD and SSD (teal/ochre). The position of the extracellular (EC) and intracellular (IC) membrane leaflets are indicated in gray. (**C** to **E**) CG potential of mean force (PMF) profiles for movement of free cholesterol (purple) and SHH-cholesterol (dark blue) between the SBD and the solvent (Δ*G*_4_) (**C**), the SSD and the bulk membrane (Δ*G*_2_) (**E**), or for extraction of cholesterol from the membrane into the solvent (Δ*G*_3_) (**D**). Bayesian bootstrapping (2000 rounds) was used to estimate profile errors (gray). Stars indicate the position of the SHH-cholesterol (within PTCH1-molA) and free cholesterol (within PTCH1-molB) densities in a revised cryo-EM structure [PDB: 6RVD (*15*)].

Discerning the direction, energetics, and stoichiometries of substrate transport by membrane proteins is essential for a holistic understanding of their molecular functions. Patched1 (PTCH1) is an integral membrane protein of the vertebrate Hedgehog (HH) signaling pathway that inhibits the class F G protein–coupled receptor Smoothened (SMO) to prevent HH signaling (*1*). PTCH1 inhibition of SMO is relieved by binding of Sonic HH (SHH) to the PTCH1 extracellular domain (ECD) (Fig. 1A and fig. S1, A and B) (*2*). HH pathway dysregulation results in severe developmental defects and compounded cancer pathologies (*3*). Current U.S. Food and Drug Administration–approved drugs targeting SMO are often overcome by tumorigenic resistance (*4*); hence, alternative avenues for pharmaceutical intervention must be explored.

The exact mechanism of PTCH1-mediated SMO inhibition remains ambiguous, despite decades of biochemical and cellular studies (*1*) and a handful of recent PTCH1 structures (*5*–*13*). Current models suggest that PTCH1 may function as a cholesterol transporter to modulate the abundance of accessible membrane cholesterol available to bind and activate SMO (*14*). Support for the proposed PTCH1 transport function is provided by observations of sterol-like densities within the ECD of PTCH1 (fig. S1C) (*5*, *8*–*10*, *15*). SHH inhibits PTCH1 by inserting a C-terminally linked cholesterol moiety into the PTCH1 ECD (fig. S1), blocking a putative cholesterol transport tunnel (*15*). PTCH1 also shares homology with members of the prokaryotic resistance-nodulation-cell division (RND) family, which export drugs, metal ions, or small molecules through their ECDs (*16*). In addition, PTCH1 is homologous to the Niemann-Pick disease type-C1 (NPC1) protein, which transports low-density lipoprotein–derived cholesterol from the lysosomal lumen to the cytoplasm (*17*). Thus, establishing the energetics of PTCH1-mediated cholesterol transport is needed to assess the feasibility of proposed transport models and further dissect how PTCH1 may inhibit SMO.

Classically, membrane transporters are characterized using vesicular uptake assays to assess the substrate specificities and ion coupling requirements of transport. No such direct assay exists for PTCH1 function owing to (i) difficulties in labeling the proposed sterol substrates, (ii) challenges with separating pre- and post-transport sterol pools (i.e., they may both reside within different microdomains of the same membrane) (*18*), and (iii) problems with reconstituting functional PTCH1 owing to a requirement for CAM-related/down-regulated by oncogenes (CDO)/brother of CDO (BOC) partner proteins or specific membrane topologies (*19*). For example, PTCH1 localizes at the base of the primary cilia in a highly curved invaginated membrane called the ciliary pocket (*20*). Consequently, PTCH1 activity is usually assayed in vivo via downstream readouts such as HH target gene mRNA levels (*21*), SMO localization to the cilia (*22*), or secondary labeling with, e.g., Perfringolysin O (PFO)-based probes for distinct cholesterol pools (*9*, *18*). Thus, there is a clear need for systematic investigation of PTCH1 function at the single-molecule level in parallel with cellular readouts.

Molecular dynamics (MD) simulations enable the inspection of protein dynamics and interactions at atomic and near-atomistic resolutions [e.g., via coarse-grained (CG) methods] (*23*, *24*). Increasingly, combinatorial MD and free energy methods are used to assess whether protein interactions with the surrounding environment are energetically favorable (*25*). In silico methods can be used to obtain free energy values of ligands/lipids binding to proteins or transitioning between membrane and aqueous environments (*26*–*28*). Previous MD simulations of HH pathway components have been used to, e.g., demonstrate the stability of a cholesterol binding site in SMO (*29*), dissect allosteric coupling between SMO cholesterol binding sites (*30*), and explore PTCH1/SMO interactions with the membrane (*25*, *31*).

Here, we combine extensive MD simulations, free energy calculations, in silico mutants, and in vivo biochemical assays of PTCH1 variants to explore cholesterol transport and ion coupling by PTCH1. We find that cholesterol export is associated with a positive free energy penalty and would, thus, require coupling to an energetic input to be made favorable. We observe binding of Na^+^/K^+^ cations to a conserved site within the PTCH1 transmembrane domain (TMD) with similar affinity, which could provide the energetic impetus for cholesterol transport. Concurrently, we use simulations of Dispatched1 (DISP1), a HH pathway protein that uses the cellular Na^+^ gradient to release lipidated SHH from the membrane, to identify a mechanism of cation-driven conformational changes via wetting of an intracellular cavity. We find that similar conformational changes are induced in the structurally homologous DISP1 and PTCH1 TMDs when multiple cations are bound. The combined simulations, totaling ~1.5 ms, provide the first energetic assessment of cholesterol transport through PTCH1, illuminating the directionality and ion coupling stoichiometries of PTCH1 transport and ion-driven conformational changes within the conserved RND family TMD.

## RESULTS

### Energetic barrier for cholesterol export through PTCH1

Previous structural, biochemical, and simulation data suggest that the PTCH1 ECD can accommodate distinct cholesterol orientations, which differentially regulate PTCH1 molecular conformations (PTCH1-molA/B) (*5*, *7*, *11*). When “SHH-cholesterol” is bound (to PTCH1-molA), putative sterol transport tunnels, visible in the presence of “free cholesterol” (bound to PTCH1-molB), are collapsed (*15*). To investigate the proposed PTCH1 transport function, we constructed free energy cycles corresponding to cholesterol movement between the TMD and ECDs of PTCH1-molA and PTCH1-molB (Fig. 1). For PTCH1-molA and PTCH1-molB, each cycle is composed of a pathway directly linking the sterol-sensing domain (SSD) and the sterol-binding domain (SBD) through PTCH1 (Δ*G*_transport_ = Δ*G*_1_), as well as an indirect pathway between the same sites (composed of Δ*G*_2_, Δ*G*_3_ and Δ*G*_4_) via the surrounding environment (Fig. 1B). Thus, free energy cycles were constructed to compare how the energetic landscape changes between the PTCH1-molA and PTCH1-molB conformations as well as between the free cholesterol and SHH-cholesterol orientations, which differ by ~180°.

### The indirect pathway

We used potential of mean force (PMF) calculations with umbrella sampling and a CG force field (*32*) to compute the indirect pathway free energy calculations. The indirect pathway was modeled using three PMFs corresponding to the movement of cholesterol between the PTCH1 SSD and the bulk membrane (PMF-2: Δ*G*_2_), cholesterol movement between the membrane and the solvent (PMF-3: Δ*G*_3_), and movement of cholesterol between the solvent and the SBD of PTCH1 (PMF-4: Δ*G*_4_). In PMF-4, the SBD provides a favorable environment for cholesterol binding relative to the solvent in both PTCH1 conformations (PTCH1-molA: Δ*G*_4_ = −83 ± 3 kJ mol^−1^, PTCH1-molB: Δ*G*_4_ = −90 ± 4 kJ mol^−1^; Fig. 1C). The PMF-4 landscape within the PTCH1 SBD is flat, suggesting that cholesterol may slide along the tunnel-like cavity within the SBD without substantial energetic penalty as opposed to localizing within a well-defined binding site. This may allow cholesterol to deviate from the cryo–electron microscopy (cryo-EM)–observed SBD binding poses (starred in Fig. 1C). For PMF-3, extraction of cholesterol from its favorable hydrophobic bilayer environment into the surrounding solvent was associated with a large, positive free energy change (Δ*G*_3_ = +88 ± 1 kJ mol^−1^; Fig. 1D) comparable to previous reports (*33*, *34*). The magnitude of the Δ*G* values for PMF-4 and PMF-3 compare well, emphasizing the hydrophobicity of the PTCH1 SBD and the selection pressure on PTCH1 to stabilize cholesterol in a domain that mimics cholesterol stabilization in its native environment.

The resolution within the detergent region of existing cryo-EM PTCH1 structures limits the unambiguous assignment of sterol densities, which has led to uncertainty in modeling the orientation of cholesterol within the SSD (*5*–*11*). For this reason, in PMF-2, we compared the free energy change of cholesterol movement out of the PTCH1 SSD when the ROH bead [equivalent to the 3β-hydroxyl (3β-OH) group of cholesterol in atomistic resolution] pointed toward the extracellular leaflet headgroups (“OH-up”) or toward the bilayer midplane (“OH-down”). For the OH-up orientation, we observe a well-defined binding site with a moderate free energy change (PTCH1-molA: Δ*G*_2_ = +18 ± 3 kJ mol^−1^, PTCH1-molB: +16 ± 4 kJ mol^−1^; Fig. 1E). By contrast, there was no observable well for cholesterol binding to PTCH1 SSD in the OH-down orientation, and the profile remained approximately flat over the reaction coordinate (PTCH1-molB: +4 ± 3 kJ mol^−1^; fig. S2). From this, we conclude that the most likely cholesterol orientation within the PTCH1 SSD is the OH-up configuration (as reported in Fig. 1E), consistent with density modeled in the refined cryo-EM structure (*15*).

Using the free energy values from the indirect pathway, we obtained an overall Δ*G* value for cholesterol movement from the PTCH1 SSD to the SBD (Δ*G*_1_). For PTCH1-molA and PTCH1-molB, we calculate Δ*G*_1_ to be +23 ± 4 and +14 ± 6 kJ mol^−1^, respectively, i.e., cholesterol binding to the SSD within the TMD is substantially more favorable than binding to the extracellular SBD. Thus, if PTCH1 functions as a cholesterol exporter, then an energetic input would likely be required for the cholesterol to move from the SSD to the SBD. Similarly, the above data are also consistent with spontaneous movement of cholesterol from the PTCH1 SBD to the SSD membrane binding site, providing that a pathway is available.

### The direct pathway

**Fig. 2.**
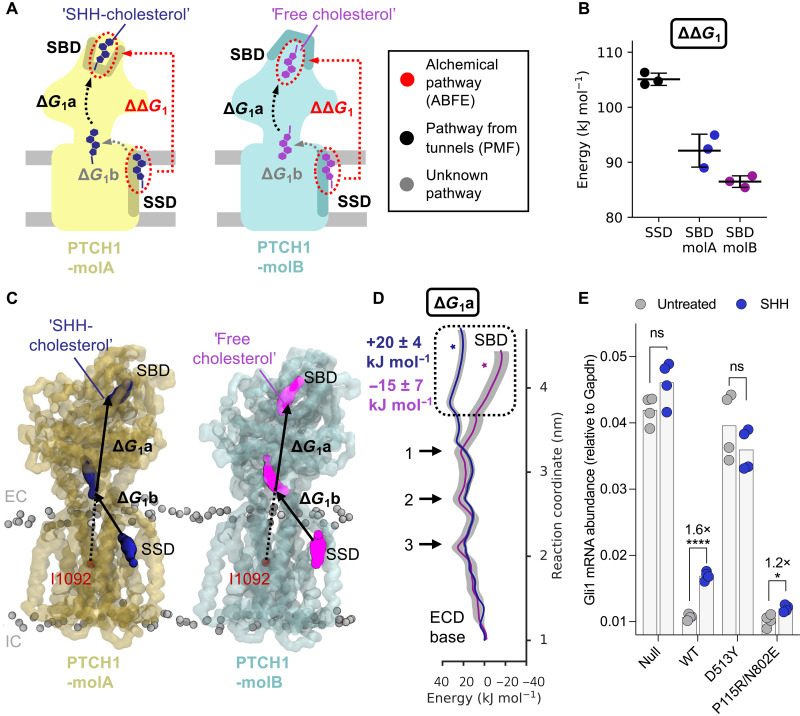
Cholesterol transport energetics—the direct pathway. (**A**) Schematic of the free energy changes associated with cholesterol movement between the PTCH1 SSD and SBD for the direct pathway, colored identically as in Fig. 1B throughout. Red dotted circles indicate alchemical decoupling of cholesterol from the SBD/SSD sites. Red dotted arrows indicate the difference between sites (ΔΔ*G*_1_). Black dotted arrows indicate movement of cholesterol between the ECD base and the SBD, used in PMF calculations to derive Δ*G*_1_a. Gray dotted arrows indicate uncertain regions of the transport pathway. (**B**) The free energy values of decoupling cholesterol from the SSD (PTCH1-molA) and SBD (PTCH1-molA and PTCH1-molB) as obtained from ABFE calculations. (**C**) Snapshots from PMF-1a for cholesterol movement between the PTCH1 ECD base and SBD (residues used in the steered MD are colored red). (**D**) PMF profile for cholesterol movement through the ECD of PTCH1-molA/B (Δ*G*_1_a) [see (C)]. Bootstrapping errors (2000 rounds) are colored gray. The position of cholesterol within the SBD pocket in the cryo-EM model [PDB: 6RVD (*15*)] is starred. Arrows in (D) indicate conserved energetic peaks within the ECD core (see fig. S4). (**E**) HH signaling strength, signified by endogenous *Gli1* mRNA abundances (normalized to the control *Gapdh)* in response to SHH ligands (200 nM, 20 hours) in *Ptch1*^−/−^ cells stably expressing PTCH1 variants. Untreated *Gli1* levels are a measure of PTCH1 activity (i.e., less *Gli1* indicates higher PTCH1 activity), while SHH-treated values represent the level of PTCH1 inactivation by SHH ligands. Statistical significance was determined by Student’s *t* test with a Welch’s correction. *P* values: *Ptch1*^−/−^ untreated versus SHH = 0.085, WT untreated versus SHH < 0.0001, D513Y untreated versus SHH = 0.2712, and P155R/N802E untreated versus SHH = 0.0205. Not significant (ns) *P* > 0.05, *P ≤ 0.05, **P ≤ 0.01, ***P ≤ 0.001, and ****P ≤ 0.0001.

Previous atomistic simulations and analysis of PTCH1 structures identified putative sterol transport tunnels throughout the PTCH1 ECD (*5*, *7*, *11*, *15*). These tunnels form between the PTCH1 SBD and cavities within the ECD in proximity to either the SSD or TM12 at the membrane interface, or side exits between PTCH1 ECD1 and ECD2. In contrast, the pathway for sterol movement between the membrane-proximal region of the PTCH1 ECD and the SSD binding site on the PTCH1 TMD is uncertain. Current predictions have not yielded a consensus pathway (*5*, *7*, *15*). Thus, while it is possible to form a PMF pathway between the SBD and the base of the ECD, using the previously identified tunnels to guide selection of an appropriate reaction coordinate, elucidation of a plausible pathway between the ECD base and the SSD TM site is nontrivial. For this reason, we used a combination of absolute binding free energy (ABFE) calculations (*35*) to directly probe the free energy difference between cholesterol bound to the SBD and the SSD sites, as well as PMF calculations confined between the SBD and ECD base to understand the energetic landscape over this subsection of the direct pathway (Fig. 2A).

**Fig. 3.**
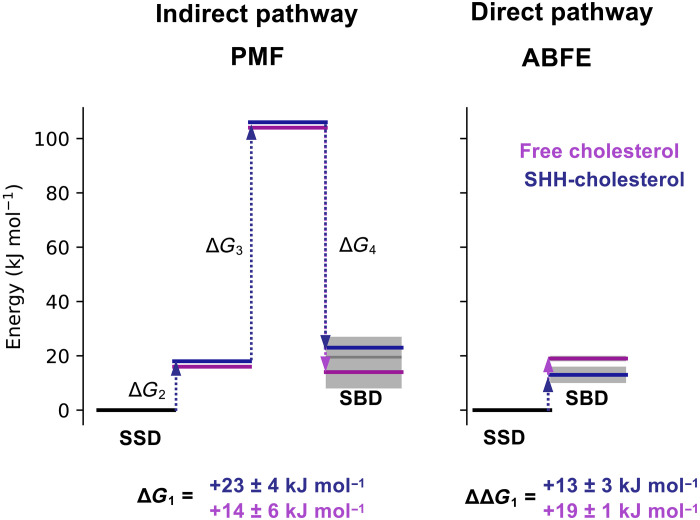
Movement of cholesterol between the SSD and SBD is associated with an energetic barrier. Comparison of Δ*G*_1_ values for free cholesterol (purple) and SHH-cholesterol (dark blue) movement between the PTCH1 SSD and SBD, derived from the indirect (PMF; Fig. 1) and direct (ABFE; Fig. 2) pathways. Stepwise free energy changes are shown as connecting dotted arrows and are labeled. Δ*G*_1_ from the indirect pathway was calculated in quadrature (Eq. 2) because PMF-2 to PMF-4 were independent. In all cases, cholesterol movement from the SSD to the SBD gives Δ*G* > 0 kJ mol^−1^.

To obtain a second estimate of Δ*G*_1_, we applied CG ABFE calculations to directly probe the free energy difference between the SHH-cholesterol (in PTCH1-molA) and free cholesterol (in PTCH1-molB) by alchemically decoupling cholesterol from the SSD and SBD of each PTCH1 conformation (Fig. 2B). The difference between decoupling from the SSD and SBD sites can be used to obtain an estimate of Δ*G*_1_ (or ΔΔ*G*_1_ as is the case here, where ΔΔ*G*_1_ = Δ*G*_1-SSD_ – Δ*G*_1-SBD_) that does not rely on formation of a physical pathway through PTCH1 (Fig. 2A). Our ABFE calculations predict a positive free energy change between the SSD and SBD sites for both PTCH1 molecules via the direct alchemical pathway, i.e., cholesterol binding to the SSD is more favorable than the SBD (PTCH1-molA: ΔΔ*G*_1_ = +13 ± 3 kJ mol^−1^, PTCH1-molB: ΔΔ*G*_1_ = +19 ± 1 kJ mol^−1^; Fig. 2B). While the magnitude of the direct pathway free energy change is reversed for PTCH1-molA and PTCH1-molB compared to the indirect pathway calculations, these data are consistent with a predicted increase in free energy between the SSD and SBD sites (Fig. 3) and therefore support the proposed hypothesis that coupling to an energy source would be required for PTCH1 to export cholesterol.

We performed PMFs of moving cholesterol between the SBD and the base of the ECD (PMF-1a: Δ*G*_1_a) to better understand the energetic landscape over the most well-defined bisection of the proposed sterol transport tunnel (Fig. 2C and fig. S3A). PTCH1-molA and PTCH1-molB show a similar energetic profile for cholesterol movement through the lower and middle regions of the ECD, consisting of three peaks of approximately +18 to +27 kJ mol^−1^ (Fig. 2D). This is suggestive of a “ratchet-like” transport mechanism formed by multiple, similar binding sites within the ECD. Overlay of the profiles in this region was confirmed by performing ABFE calculations of the two cholesterol molecules bound within a central portion of the ECD (2.4 nm on PMF-1a), which yielded similar Δ*G* values (PTCH1-molA: +50 ± 1 kJ mol^−1^, PTCH1-molB: +55 ± 2 kJ mol^−1^). Notably, as cholesterol moves toward the SBD, the paths for PTCH1-molA and PTCH1-molB diverge. Cholesterol movement toward the cryo-EM binding pose within the SBD of PTCH1-molB (purple star in Fig. 2D) is associated with a stabilization of −15 ± 7 kJ mol^−1^ compared to the free energy at the ECD base (1 nm in Fig. 2D). Conversely, movement of cholesterol toward the PTCH1-molA SBD pose (dark blue star in Fig. 2D) is associated with an increase in free energy of +20 ± 4 kJ mol^−1^. This is consistent with previous atomistic simulations that show a stabilization of the ECD upper lobe by cholesterol in PTCH1-molB but not PTCH1-molA, as demonstrated by a reduced root mean square deviation (RMSD) compared to the apo ECD conformations (*15*). This result also corroborates our PMF-4 profiles that suggest that the PTCH1-molB SBD cholesterol binding site is stabilized relative to PTCH1-molA, albeit to a lesser extent. Furthermore, in all PTCH1 structures solved to date with a SBD conformation matching that of PTCH1-molB, sterol-like density is observed in an equivalent region to the free cholesterol molecule (fig. S1C) (*5*, *8*–*10*). Together with our observations of a free energy well toward the PTCH1-molB SBD, this suggests that the free cholesterol site may represent a local binding site within the ECD, perhaps facilitating downhill (i.e., favorable) movement of cholesterol toward the SBD to promote transport directionality.

Because of the uncertainties in the transport pathway between the SSD and the ECD base (described previously), it is not possible to obtain full characterization of the energetic landscape using PMF calculations over the membrane-proximal region of PTCH1. This is complicated by the requirement for the free cholesterol to flip between the ECD base and SSD sites in PTCH1-molB, potentiating difficulties with obtaining this PMF (Fig. 2A). Lipid reorientation is also observed in other RND proteins such as in MmpL3 (*36*). Our attempts (PMF-1b: Δ*G*_1_b), confined to fig. S3 (B and C), show a large energetic barrier that occupies the central region of the PMF-1b profile. This occurs as a result of solvation of the hydrophobic cholesterol molecule as it leaves the tunnel cavity at the base of the ECD and slides along the protein surface toward the bilayer. It seems unlikely that this represents a physiological pathway considering the efficiency of HH signaling pathway inhibition by PTCH1 and the high insolubility of cholesterol and, hence, is excluded from our predictions of Δ*G*_1_. In summary, our Δ*G*_1_ values calculated from the indirect (PMF) and direct (ABFE) pathways both suggest moderate energetic costs for cholesterol movement from the SSD to the SBD of approximately +13 to +23 kJ mol^−1^ (Fig. 3).

### Blocking the ECD sterol tunnel does not disrupt PTCH1 function

We sought to identify those residues that may contribute to energetic bottlenecks in the cholesterol pathway through PTCH1 ECD and that may therefore provide insight into plausible therapeutic interventions. We identified residues within 0.6 nm of cholesterol in those windows that surround energetic peaks in the PMF-1a profile (Fig. 2D, see marked arrows). The four residues with the highest occupancy, i.e., residues that are within 0.6 nm of cholesterol for the greatest fraction of the simulation time, were mapped onto the structure of PTCH1 at each peak (fig. S4A). The residues localize to two layers at the ECD1-ECD2 interface, which we term the upper and lower restrictions. Visualization of the PMF window trajectories suggests that cholesterol interaction with the upper and lower restrictions results in the first and third energetic peaks, respectively, whereas the middle peak is formed by cholesterol interacting with both the lower and the upper restrictions simultaneously. We also calculated the per residue cholesterol occupancies over all PMF-1a windows, which showed that residues comprising the upper and lower restrictions also had the highest occupancies over the whole PMF-1a profile (fig. S4B). There is good agreement between identified residues from PTCH1-molA and PTCH1-molB because of conservation of the energetic profile over the lower and middle regions of the ECD (Fig. 2D). In particular, in PTCH1 molecules, L227, M335, W337, A936, and Q938 contribute to the upper restriction just below the base of the SBD pocket, and P155 and N802 form part of the lower restriction just above a previously identified sterol site (*9*) near the entrance to the putative sterol conduit at the base of the ECD.

Observed sterol densities in PTCH1 structures do not necessarily indicate cholesterol movement between these sites (or within the internal PTCH1 ECD cavity we probed computationally). Sterol detergents added during protein purification (e.g., CHS) may bind to distinct sites without interconnected sterol translocation through the ECD cavity. Given the +18 to 27 kJ mol^−1^ energetic penalty associated with cholesterol movement through the lower and upper restrictions (Fig. 2D), we sought to assess whether cholesterol movement through the ECD is necessary for PTCH1 function in cells. To further dissect whether proposed sites represent distinct cholesterol binding sites or a genuine interconnected cholesterol pathway, we generated a salt bridge mutant (P155R/N802E) that occludes the proposed PTCH1 ECD transport cavity (Fig. 2E and fig. S5). We generated cell lines stably expressing PTCH1 variants in a *Ptch1* knockout background to cleanly assess their ability to inhibit HH signaling. As expected, cells lacking *Ptch1* (*Ptch1*^−/−^) show high basal HH strength because of unrestrained SMO signaling downstream (Fig. 2E). Expression of either wild-type (WT) PTCH1 or the P155R/N802E mutant blocked SMO activity and reduced HH signaling strength, demonstrating that these proteins are active. As a control, we expressed an inactive PTCH1 mutant, D513Y, which was unable to block HH signaling in the *Ptch1*^−/−^ background. The comparable activity of WT PTCH1 and the P155R/N802E mutant suggests that either (i) introducing a salt bridge to the cholesterol tunnel in PTCH1 does not block its function or (ii) this mutation is not sufficient to block cholesterol movement through the tunnel. Next, we tested the ability of SHH ligands to inhibit PTCH1 activity. The P155R/N802E mutant showed reduced responsiveness to SHH ligands compared to WT PTCH1. This suggests that the predicted salt bridge forms and disrupts SHH binding, without disrupting PTCH1 activity. Hence, we provide cellular evidence that sterol translocation through the ECD may not be required for PTCH1 function, in agreement with previous observations that PTCH1 can still function to inhibit SMO in the absence of the whole ECD2 domain (*37*, *38*).

### Identification of multiple, interconnected cation binding sites within PTCH1 TMD

PTCH1 shares a conserved structural architecture with members of the prokaryotic RND transporter superfamily, which utilize the proton gradient to export small molecules, drugs, peptides, and metals (*16*, *39*). The proposed RND proton binding site is formed by two or three anionic residues in the center of the TMD (*40*). PTCH1 sequence alignment has identified two aspartate residues (D513 and D514) on TM4 and a glutamate residue on TM10 (E1095) as forming part of highly conserved GXXXDD and GXXX(E/D) motifs at the center of the TMD, in equivalent positions to the proton binding sites in RND transporters (*5*, *9*).

**Fig. 4.**
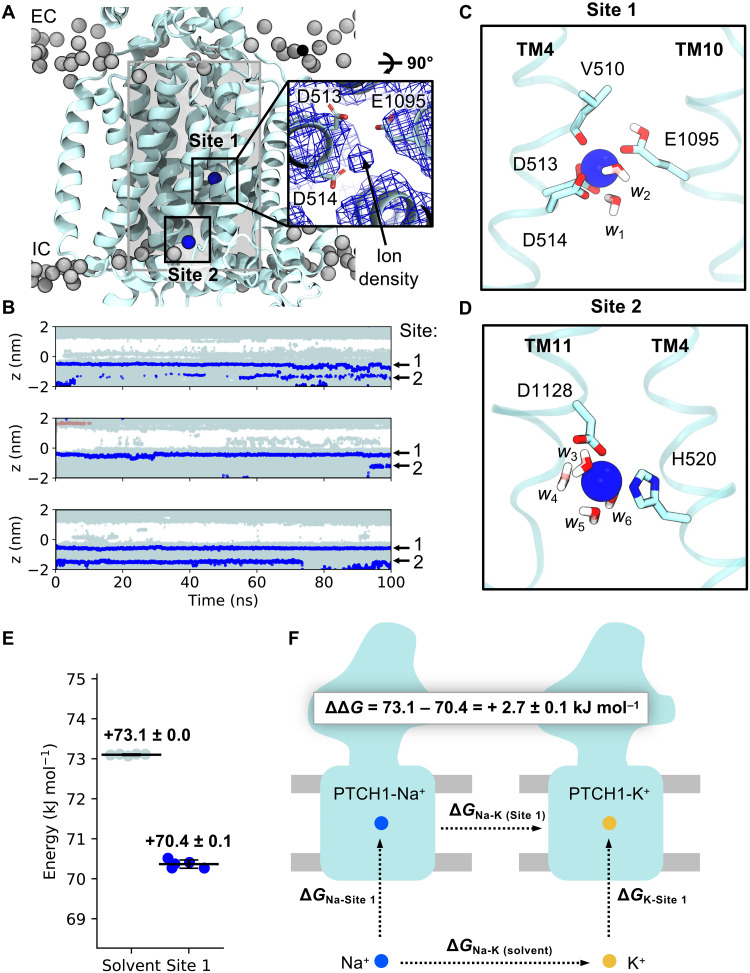
Identification and selectivity of cation binding sites within the PTCH1 TMD. (**A**) Snapshot from atomistic simulations of PTCH1 [light blue; PDB: 6DMY (*5*)] indicating the location of cation binding sites within the TMD. Na^+^ ions are shown as blue spheres and lipid phosphates are gray. Inset: Cation-like density surrounded by anionic tired residues at site 1 within the cryo-EM structure (*5*), as viewed from the extracellular face. A cylinder (length of 4 nm, radius of 1.3 nm) centered on the midpoint of V510 and I1092 Cα atoms is shown in gray and was used to identify water and ions within the PTCH1 TMD (B). (**B**) The *z* coordinates of water oxygen atoms (light blue), Na^+^ (blue), and Cl^−^ (salmon) ions localized within the PTCH1 TMD [see (A); gray cylinder] over the length of 3 × 100 ns of simulations initiated with Na^+^ bound at the density observed in (A). Snapshots of site 1 (**C**) and site 2 (**D**) showing coordination of bound Na^+^ ions by surrounding residues (stick representation) or waters (*w*_1_ to *w*_6_). (**E**) Free energy perturbation (FEP) calculations for alchemical transformation of Na^+^ into K^+^ within the solvent or bound to site 1. (**F**) Schematic representation of the free energy cycle used to calculate the difference in Na^+^ binding to site 1 compared to K^+^ (ΔΔ*G*).

We previously identified an ion-like density in proximity to the anionic triad within an existing PTCH1 structure (Fig. 4A, inset) (*5*, *15*). Given our finding that cholesterol movement between the SSD and SBD is associated with a free energy barrier, we reasoned that cholesterol export could be made favorable by coupling to cation binding and/or movement across the membrane.

To test the viability of the proposed cation binding site, we performed atomistic simulations of PTCH1 with Na^+^ located in the TMD according to the cryo-EM density at a position we term site 1 (Fig. 4). Na^+^ remained bound at site 1 over the 100 ns of three repeat trajectories with a mean RMSD of 0.15 ± 0.02 nm compared to the initial ion position (Fig. 4B). There is active debate within the PTCH1 literature on the molecular identity of the proposed coupling cation, with both Na^+^ and K^+^ proposedly implicated in PTCH1 function or regulation (*21*, *22*, *41*). Hence, we performed further simulations, initiated with an apo TMD (in 0.15 M NaCl or KCl) and observed spontaneous Na^+^/K^+^ binding to site 1 in all replicates (fig. S6A). Na^+^/K^+^ were coordinated octahedrally by six oxygen atoms from the carboxyl groups of D513^−1^, D514^−1^, and E1095^0^ (where the superscript indicates the modeled charge of the residue); the backbone carbonyl of V510; and two water molecules (Fig. 4C). Residue-ion contact mapping showed occasional displacement of the water at position *w*_2_ by the hydroxy side chain of T551 (Table 1). Application of a membrane voltage of physiological magnitude was not sufficient to promote displacement of Na^+^ from site 1 (fig. S6B). A second cation binding site (site 2; Fig. 4), not visible in the cryo-EM data, was observed in the simulation data on TM4/TM11 (Fig. 4D), with an ion from bulk solvent entering a small solvent–accessible cavity formed by local bilayer deformation at the intracellular PTCH1 surface [also visible in MemProtMD (*42*)]. Here, Na^+^/K^+^ is coordinated by four water molecules and two residues from the TMD, H520^0^ and D1128^−1^. A similar solvated cavity was observed in simulations of the RND member AcrB, whereby a continuous solvent pathway extends from this surface cavity into the TMD core (*43*, *44*). In some simulations, Na^+^/K^+^ was observed to move from site 2 into site 1 mediated by flipping of D1128 toward TM4 to hand over Na^+^ to D513, even when site 1 was already occupied. This suggests that site 2 might act as an intermediate site between the solvent and site 1 and that anionic triad residues are capable of coordinating at least two cations simultaneously.

**Table 1.** Residue contacts with Na^+^ bound at site 1 across simulations (3 × 100 ns) of PTCH1. Residues with <1% contact are excluded.

Residue	No. frames within 0.3 nm of Na^+^ (%)
D514	93.3
D513	89.5
V510	85.1
E1095	81.8
T551	5.8

The suggested involvement of K^+^ in PTCH1 function (*21*, *41*) is unexpected given an absence of known eukaryotic K^+^ coupled transporters and the theoretical closeness of the resting plasma membrane potential and the equilibrium K^+^ potential (*41*). To explore this further, we calculated the free energy associated with perturbation of Na^+^ to K^+^ at site 1 compared to bulk solution using free energy perturbation (FEP) calculations (Fig. 4, E and F, and fig. S7). Perturbation of Na^+^ to K^+^ was a marginal 2.7 ± 0.1 kJ mol^−1^ more favorable when bound to site 1 than in solution, suggesting that Na^+^/K^+^ binds to site 1 with very similar affinity. Thus, it is perhaps expected that both ions have been implicated in PTCH1 function/regulation biochemically (*21*, *41*).

### An intracellular open solvent cavity forms within the PTCH1 TMD

**Fig. 5.**
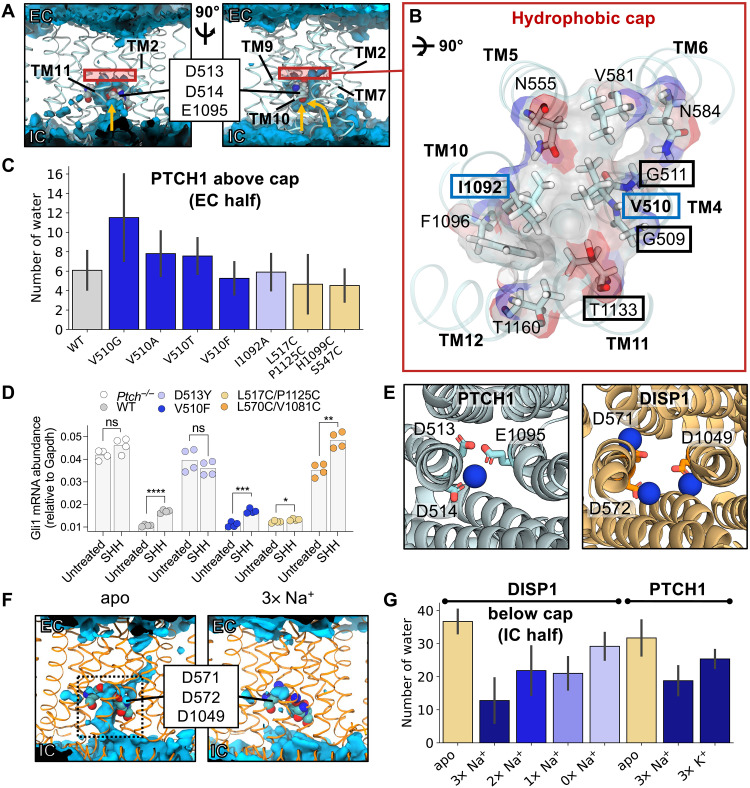
Identification of “inward-open” and solvent “occluded” PTCH1/DISP1 conformations. (**A**) Time-averaged water density (blue isosurface) across a 100-ns simulation of PTCH1 [PDB: 6DMY (*5*)] initiated with Na^+^ bound at site 1. PTCH1 TMD is shown as ribbons, and anionic triad residues are spheres. Yellow arrows indicate paths for water entry. (**B**) Residues comprising the hydrophobic cap [red box in (A)], viewed from the extracellular (EC) face. V510 and I1092 [within the conserved GXXXDD and GXXX(E/D) motifs] are boxed in blue. Residues mutated in disease phenotypes are boxed in black. (**C**) Mean number of waters per frame within the TMD EC portion across the final 10 ns of 3 × 50 ns of simulations of WT PTCH1 or PTCH1 mutants (see the Supplementary Materials). (**D**) HH signaling strength, signified by endogenous *Gli1* mRNA abundances in *Ptch1*^−/−^ cells stably expressing PTCH1 variants (see Fig. 2E for details). *P* values: *Ptch1*^−/−^ untreated versus SHH = 0.085, WT untreated versus SHH < 0.0001, D513Y untreated versus SHH = 0.2712, V510F untreated versus SHH = 0.0002, L517C/P1125C untreated versus SHH = 0.0255, and L570C/V1081C untreated versus SHH = 0.0014. Not significant (ns) *P* > 0.05, *P ≤ 0.05, **P ≤ 0.01, ***P ≤ 0.001, and ****P ≤ 0.0001. (**E**) Comparison of cation binding sites within PTCH1 and DISP1 [PDB: 7RPH (*46*)] TMDs. (**F**) Time-averaged water density profiles across 100-ns simulations of DISP1 in apo or “3× Na^+^” bound conformations. (**G**) Mean number of waters per frame within the intracellular (IC) half of the DISP1 TMD across the final 10 ns of 3 × 100 ns of simulations of DISP1 in apo and 3× Na^+^ bound states, or in 2× Na^+^, 1× Na^+^, and 0× Na^+^ bound states generated by sequential ion removal from the end of the previous Na^+^-bound state or PTCH1 in apo, 3× Na^+^, and 3× K^+^ bound states (see the Supplementary Materials). (C) and (G) report the mean and SD between repeats.

To gain insight into the role of the PTCH1 TMD, we analyzed the TMD localized water density across atomistic simulations of PTCH1. A hydrated cavity encompassing the anionic triad residues and opening to the bulk intracellular solvent was clearly visible from both the water density analysis and the *z* coordinates of water and ions over time (Figs. 4B and 5A and fig. S6). Water entered the cavity between TM4 and TM10 at the base of the TMD or via the water pocket surrounding site 2 (Fig. 5A, see arrows). The extracellular TMD half remained dewetted, and water did not cross between the extracellular and intracellular compartments during the simulations, akin to previously observed water networks present in the O and L states of AcrB (*43*).

### Identification and mutation of the hydrophobic cap

Trajectory visualization suggested that water permeation into the extracellular region of the TMD may be prevented by several hydrophobic residues situated directly above the anionic triad (Fig. 5B). Notably, two residues involved in the formation of the hydrophobic cap, V510 and I1092, are each flanked by two glycine residues that could facilitate side chain swivel to enable solvent permeation. These residues also comprise the conserved GXXXDD and GXXX(D/E) motifs on TM4 and TM10. To probe this hypothesis, we performed atomistic simulations of WT PTCH1 and PTCH1 TMD mutants without Na^+^ initially bound at site 1. Residues constituting the hydrophobic cap (V510 and I1092) were selected for in silico mutation in an attempt to promote solvent permeation through the TMD. In 14/18 (78%) of trajectories, Na^+^ entered the TMD and bound at site 1, often within the first 20 ns of the simulations (fig. S6). We analyzed the number of waters within the extracellular TMD half of a cylinder surrounding residues V510 and I1092 within the last 10 ns of each simulation (Fig. 5C). Compared to WT PTCH1 (waters: 6 ± 2), the average number of water molecules per frame within the extracellular half of the TMD was increased when V510 was mutated to glycine (waters: 12 ± 5) but not when mutated to a smaller hydrophobic (V510A; waters: 8 ± 2) or polar (V510T; waters: 8 ± 1) residue. The I1092A (waters: 6 ± 1) mutation also had no effect on the solvation of the extracellular half of the TMD nor did attempts to disrupt the packing of cap residues (V510F; waters: 5 ± 1). To functionally test the role of the V510 residue in forming a hydrophobic cap, we tested the V510F mutation in HH signaling assays. In cells lacking *Ptch1* (*Ptch1*^−/−^), high levels of unrestrained HH signaling are observed (Fig. 5D) compared to the repressed SMO activity in cells expressing WT human Ptch1 (hPTCH1). Mutation of D513Y, proposed to coordinate Na^+^/K^+^ ion binding at site 1 in PTCH1, was not functional to inhibit HH signaling. Next, we tested the activity of V510F and found that it is functional to block HH signaling, and it demonstrates responsiveness to SHH ligand addition. This in vivo mutational data are consistent with the in silico data showing that water permeation was unaltered by the V510F mutation. We also expressed the V510G mutant in *Ptch1^−/−^* cells, predicted to have increased water permeation into PTCH1, but this mutant displayed lower expression by Western blot analysis (fig. S5). This may suggest that increased solvent permeability in PTCH1 reduces its stability.

Overall, our in silico and in vivo data suggest that a model for solvent permeation governed purely by a simple hydrophobic restriction at one position on TM4/TM10 (as occurs in, e.g., ion channels) is overly simplistic. This suggests that ion coupling likely involves more complex concerted TMD motions.

### Breathing-like motions of the PTCH1 TMD mediate ion binding and PTCH1 function

We next tested whether mutations at the intracellular TMD face of PTCH1 could block solvent permeation. We tested the effect of introducing disulfide bonds at residues near the opening of the hydrophilic cavity to sterically prevent water entry. First, we cross-linked the intracellular TMD face on TM4 to TM11 (L517C to P1125C; waters: 4 ± 3) or TM5 to TM10 (H1099C to S547C; waters: 4 ± 1), which marginally decreased water permeation and, notably, prevented Na^+^ entry to TMD site 1 in all but one trajectory (Fig. 5C and fig. S6). These cross-linked mutants support the proposal that breathing-like motions of the intracellular TMD regions, restricted in the cross-linked mutants, may regulate ion entry within PTCH1.

To further dissect the role of TMD motions in PTCH1 function, we performed in vivo assays of PTCH1 mutants (Fig. 5D). Using the system described previously, we expressed L517C to P1125C in *Ptch^−/−^* cells and found that it was functional to inhibit SMO activity. This mutant was not responsive to SHH ligands, suggesting that conformational flexibility at the intracellular side of PTCH1 is required for its inactivation by SHH. Alternatively, the disulfide bond expected to form between the two residues may not occur when PTCH1 is in an active conformation with the open intracellular cavity, but may form in a conformational intermediate induced by SHH binding. Next, we tested a disulfide mutant on the extracellular side of the TMD, L570C/V1081C. Unexpectedly, this mutant was not functional, despite being expressed at high levels, but did exhibit SHH responsiveness (Fig. 5D and fig. S5). This suggests that conformational flexibility in the extracellular face of the TMD is required for PTCH1 function. This type of movement may be required for ion movement from outside the cell toward the pocket or for release of ions within the pocket to the extracellular aqueous environment. Future work is needed to distinguish between these models.

### Insights from DISP1: Na^+^ binding induces transition to an occluded conformation

DISP1 is an RND transporter that catalyzes the Na^+^ coupled export of SHH from the plasma membrane into the extracellular space (upstream of SHH engagement with PTCH1) (*21*, *45*, *46*). The DISP1 TMD shares homology with PTCH1 (TMD backbone RMSD: 0.22 nm), and a recent DISP1 structure revealed three Na^+^ ions bound to the anionic triad within the TMD (Fig. 5E) (*46*). We performed atomistic simulations of DISP1 in “3× Na^+^” and apo states to assess (i) whether three Na^+^ ions could be accommodated within this small, charge-dense region and (ii) whether DISP1 could inform our understanding of ion coupling in PTCH1 function. Analysis of the time-averaged water density within apo DISP1 revealed a solvated intracellular open cavity, similar to PTCH1 (Fig. 5F). Unexpectedly, this intracellular cavity was absent in the 3× Na^+^ state, and all three Na^+^ ions were stably coordinated within the TMD (RMSD: 0.16 ± 0.01 nm per Na^+^), validating the interpretation of cryo-EM densities (*46*). We reasoned that the 3× Na^+^ ions may stabilize a solvent occluded state by interlocking neighboring TM helices. To test this, TMD Na^+^ ions were sequentially removed from the end snapshot of simulations to generate successive 2× Na^+^, 1× Na^+^, and 0× Na^+^ states. Cation removal increased the average number of waters per frame within the intracellular region of the TMD, indicating that all three Na^+^ ions contribute to TMD stabilization and restriction of water entry (Fig. 5G).

**Fig. 6.**
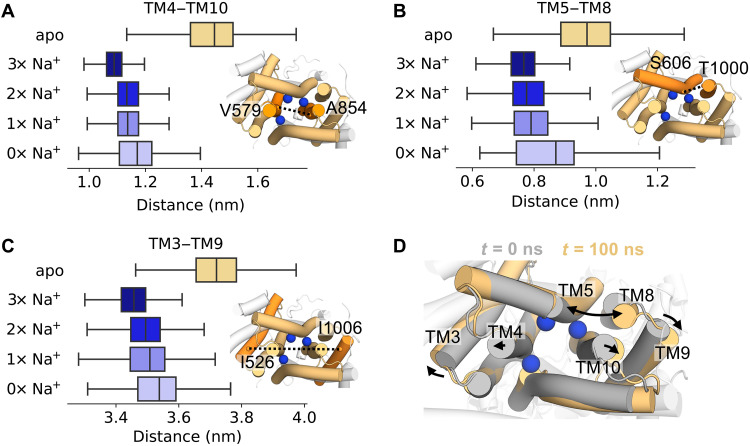
Na^+^ interdigitates the DISP1 intracellular TMD helices. (**A** to **C**) Minimum distances between the intracellular portions of DISP1 helices across 3 × 100 ns of simulations of DISP1 in apo, 3× Na^+^, 2× Na^+^, 1× Na^+^, and 0× Na^+^ bound states. Distances were calculated between the Cα atoms of labeled residues on (A) TM4 to TM10, (B) TM5 to TM8, and (C) TM3 to TM9. Snapshots of the DISP1 TMD from the intracellular face are shown, with black dotted lines marking the distance between helices (orange). (**D**) Expansion of the intracellular TMD helices as indicated by comparison of the start (gray) and the end (yellow) snapshots from a simulation of apo DISP1. The position of Na^+^ ions (blue spheres) are overlaid for reference.

We calculated minimum distances between the intracellular portions of TM helices in DISP1. Compared to extracellular TM distances (which remained within ~0.1 nm regardless of Na^+^ bound state; fig. S8A), distances between intracellular TM portions were constantly increased in the apo state by ~0.4 nm relative to the 3× Na^+^ state (Fig. 6). Sequential removal of ions increased intracellular TM distances toward those of the apo state. Thus, 3× Na^+^ ion binding within the TMD of DISP1 facilitates transition from an “inward-open” to solvent “occluded” state by restricting outward displacement of the intracellular regions of TM helices (Fig. 6D).

Propelled by these findings, we sought to assess whether cation-mediated TMD interdigitation also occurs in PTCH1. Because the PTCH1 and DISP1 TMDs are structurally homologous (TMD backbone RMSD: 0.22 nm), we reasoned that the presence of multiple cations may induce changes pertinent to PTCH1 function. We simulated PTCH1 with either 3× Na^+^ or 3× K^+^ ions bound at equivalent positions in the DISP1 structure (*46*). Na^+^/K^+^ ions remained bound albeit with a higher RMSD (0.33 ± 0.03 nm per Na^+^, 0.43 ± 0.05 nm per K^+^) than observed for DISP1 Na^+^ ions, which may indicate that further side chain rearrangements are required for optimal coordination. For example, exchange with or movement toward site 2 was occasionally observed for the Na^+^/K^+^ ion coordinated by D513 (equivalent to DISP1 D571) (fig. S6). As in DISP1, the presence of 3× Na^+^/K^+^ ions reduced water entry into the PTCH1 TMD cavity compared to apo (Fig. 5G) and prevented TMD opening between occluded (3× Na^+^/K^+^) and inward-open (apo or 1× Na^+^ bound) conformations (fig. S8, B and C). This corroborates our findings from the cross-linked in silico and in vivo PTCH1 mutants that subtle breathing-like motions play a role in solvent permeation through the TMD and may also explain why ordered, trapped water molecules could be resolved with the DISP1 TMD (*46*) but not in current PTCH1 structures.

## DISCUSSION

**Fig. 7.**
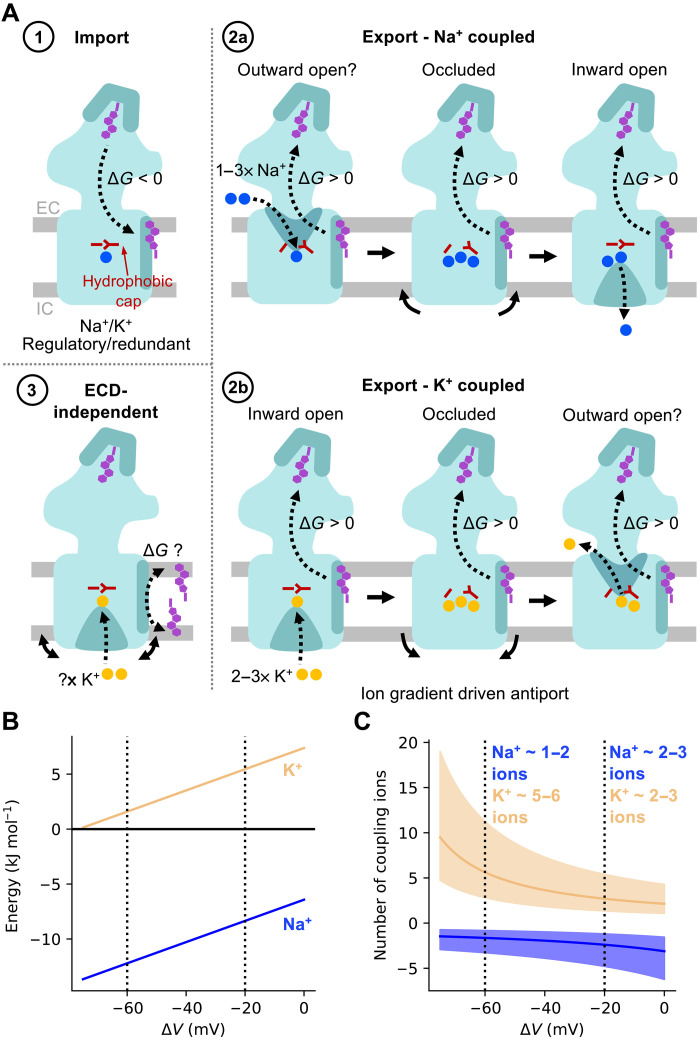
Energetics and ion coupling of PTCH1 transport models. (**A**) Proposed models for PTCH1 function (colored as in Fig. 1). Model 1: PTCH1 imports cholesterol extracted from SMO to a membrane sequestered pool or intracellular donor (energetically favorable). Model 2: Accessible cholesterol export by PTCH1 mediated by coupling to one to three Na^+^ ions (blue; model 2a) or two to three K^+^ ions (yellow; model 2b). Transition between inward-open and occluded states accompanied by breathing-like motions of intracellular helical segments (solid arrows) and alleviation of the hydrophobic cap (red). Model 3: PTCH1 repartitions accessible cholesterol to an intramembrane sequestered pool (e.g., partnered with sphingomyelin) or intracellular acceptor. (**B**) Free energy stored across the Na^+^ (blue) and K^+^ (yellow) membrane gradients as a function of membrane potential (Δ*V*) (Eq. 1). (**C**) Predicted number of Na^+^/K^+^ coupling ions required per cholesterol exported by PTCH1 [defined as “cholesterol export free energy”/”free energy across cation potential” from (B)] versus membrane potential (Δ*V*). A cholesterol export free energy of +20 kJ mol^−1^ is indicated by a solid line, with export energies ranging between +10 to +40 kJ mol^−1^ indicated in transparent. Standard cellular ion concentrations ([Na^+^]_in_: 12 mM, [Na^+^]_out_: 145 mM, [K^+^]_in_: 150 mM, [K^+^]_out_: 4 mM) are assumed.

Several models have been proposed for PTCH1-mediated SMO inhibition (Fig. 7A), extending from biochemical observations, suggesting PTCH1 reduces accessible cholesterol levels in the cilia and, thus, lowers SMO activation levels (*1*, *14*). Models include extraction of cholesterol from the SMO cysteine-rich domain and import through PTCH1 to a sequestered cholesterol pool (such as in complex with sphingomyelin) or intracellular acceptor (model 1), export of accessible cholesterol through PTCH1 (model 2), or repartitioning of accessible cholesterol via an ECD-dependent mechanism (model 3) (*14*, *41*).

Using two independent free energy methods, we predict cholesterol import (model 1) to be energetically favorable, whereas movement in the export direction (model 2) would require coupling to an energy source. Model 1 is not consistent with the requirement of cations for PTCH1 function (*21*, *22*, *41*) or with anionic triad mutants that render PTCH1 less functional and are associated with severe co-occurrences of Gorlin’s syndrome (*5*, *37*, *47*–*49*). There are also no known extracellular cholesterol shuttles between SMO and PTCH1 that would presumably be necessary given their distinct membrane localizations (*20*, *50*). The PTCH1 homolog NPC1 (*51*–*55*) can, however, spontaneously transport cholesterol in the import direction (*56*), which can be rationalized by our free energy calculations.

Our atomistic simulations suggest that cholesterol export could be made favorable by coupling to translocation of Na^+^/K^+^ through the TMD (Fig. 4 and figs. S6 and S7). Biochemically, both Na^+^ (*22*) and K^+^ (*21*, *41*) have been implicated in PTCH1 function; however, assay interpretation is complicated by use of downstream signaling readouts for PTCH1 activity. K^+^ involvement is also perplexing given that there are no known eukaryotic K^+^-coupled transporters, and the K^+^ equilibrium potential approaches the predicted plasma membrane potential. Accordingly, our FEP calculations predict similar binding affinities for K^+^ and Na^+^ at site 1 (Fig. 4, E and F). To estimate the cholesterol:cation transport stoichiometry, we calculated the free energy stored in the Na^+^/K^+^ gradient as a function of membrane potential (Fig. 7B and Eq. 1). This was used to obtain the number of export-coupled cations, assuming a generous cholesterol export free energy of Δ*G*_1_ = +10 to +40 kJ mol^−1^ and standard cellular ion concentrations ([Na^+^]_in_: 12 mM, [Na^+^]_out_: 145 mM, [K^+^]_in_: 150 mM, [K^+^]_out_: 4 mM) (Fig. 7C).

$$\text{Δ}G=\mathrm{RTln}\left(\frac{{\left[X^{+}\right]}_{\mathrm{in}}}{{\left[X^{+}\right]}_{\mathrm{out}}}\right)+zF\text{Δ}V$$ (1)

Thus, we predict one to three Na^+^ (model 2a) or two to three K^+^ (model 2b) ions to be required per cholesterol transported, although K^+^-driven transport could only occur if the cilia membrane potential was less electronegative than expected (approaching −20 mV) (Fig. 7C). There is some suggestion that this may be the case (*57*); however, it is unclear how this would be established given that the cilia is contiguous with the plasma membrane.

While a PTCH1 cholesterol export model is supported by ECD localized sterol densities in cryo-EM structures (*5*, *8*–*10*), it is difficult to reconcile with one long-standing observation; PTCH1 ECD2 cleavage renders PTCH1 constitutively active (*37*, *38*). A recently proposed ECD-independent mechanism (model 3) addresses this by suggesting that PTCH1 may promote repartitioning of accessible cholesterol to an intracellular acceptor or sphingomyelin sequestered pool (*41*). Hence, the ECD may simply be a socket for SHH engagement independent of PTCH1 function. Our free energy calculations support a role for the SBD as an energetic switch between SHH-cholesterol– and free cholesterol–engaged conformations (Fig. 2D). The free energy of interleaflet cholesterol flippase activity would presumably be much lower [given that this occurs spontaneously in membranes (*58*)] and, thus, may be more amenable to reduced free energy stored in the K^+^ potential at ~−60 mV. Thus, we turn the export argument on its head by asking whether the incompatibility of the K^+^ and cholesterol export free energies (at ~−60 mV; Fig. 7, B and C) can be used as further evidence against an ECD-dependent PTCH1 function. This is consistent with our data for the ECD salt bridge (P115R/N802E), retaining its ability to block HH signaling while losing SHH responsiveness (Fig. 2E).

Last, we complemented our PTCH1 analyses with simulations of DISP1 in distinct Na^+^-bound states (Figs. 5 and 6) (*46*). In the 3× Na^+^ state, the solvent is prevented from entering DISP1 by Na^+^-mediated interdigitation of the intracellular portions of TM helices. Ion removal promoted transition from a solvent occluded to inward-open conformation by concerted outward movement of intracellular helical segments (Fig. 6). This effect was also observed when PTCH1 was simulated in the presence of 3× Na^+^/K^+^ ions (Fig. 5 and fig. S8). Thus, we provide the first molecular description of the conformational transition between distinct ion-coupled transport states of DISP1/PTCH1. More work is needed to establish the events leading to putative “outward-open” conformations and/or cross-membrane ion translocation. In conclusion, we provide energetic contextualization to the directionality and stoichiometry of PTCH1 transport models and elucidate conformational transitions pertinent to other RND protein mechanisms.

## MATERIALS AND METHODS

### CG PMF calculations

We previously performed CG MD simulations of PTCH1-molA and PTCH1-molB from the rebuilt PTCH1-SHH (2:1) structure [Protein Data Bank (PDB): 6RVD] embedded in symmetric 1-palmitoyl-2-oleoyl-sn-glycero-3-phosphocholine (POPC):cholesterol (CHOL) (3:1) bilayers (Fig. 1A) (*15*, *59*). Snapshots from one 10-μs simulation of PTCH1-molA and PTCH1-molB were selected for use in PMF calculations. In total, nine PMFs were run for five coordinates (see table S1). PMF-1a used full-length PTCH1-molA (with cholesterol bound to the SBD in the SHH-cholesterol orientation) or PTCH1-molB (with cholesterol bound in the free cholesterol orientation). PMF-1b was built from the final window of PMF-1a with the cholesterols positioned at the base of the ECD. PMF-4 used the PTCH1-molA and PTCH1-molB ECDs with the SHH-cholesterol and free cholesterol bound to the SBD as in PMF-1a (and according to the cryo-EM densities). PMF-3 used a 10-nm by 10-nm bilayer patch (in the absence of protein). PMF-2 was constructed as in PMF-1a but with cholesterol bound to the SSD instead of the SBD. See the Supplementary Materials for full system details including setup and cholesterol orientations.

PMF calculations were carried out in accordance with a previously published method (*60*). GROMACS 2018 and 2019 (www.gromacs.org) were used to perform all simulations (*61*). Steered MD simulations were used to pull the cholesterol of interest through the protein (PMF-1), away from the protein (PMF-2 and PMF-4), or out of the membrane (PMF-3). These followed a one-dimensional (1D) reaction coordinate between the center of mass (COM) of cholesterol and a backbone bead of PTCH1 (PMF-1, PMF-2, and PMF-4; see the Supplementary Materials) or the COM of the bilayer (PMF-3). An umbrella pulling force of 1000 kJ mol^−1^ nm^−2^ and a pulling rate of 0.1 nm ns^−1^ were used in all cases, with the exception of PMF-1b where the pulling rate was 10 nm ns^−1^ to prevent extraneous pulling of cholesterol into the aqueous solvent after egression from the ECD. Windows were spaced every 0.05 nm along the reaction coordinate and simulated for 1 to 3 μs each until convergence (table S1 and fig. S9). An umbrella potential (1000 kJ mol^−1^ nm^−2^) was used to limit cholesterol movement within each window. PMF profiles were constructed using the weighted histogram analysis method implemented in gmx wham (*62*, *63*). The first 200 ns of each window was discarded as the equilibration time, and 2000 Bayesian bootstraps were used to estimate the PMF error (PMF*^e^*). Because PMF-2 to PMF-4 are independent, the total error (*e*_T_) of the indirect pathway was calculated as in Eq. 2. Setup and analysis of PMFs were aided by use of the pmf.py tool (DOI:10.5281/zenodo.3592318) (*60*)

$$e_{T}=\sqrt{\sum_{2-4}{\left({\mathrm{PMF}}_{x}^{e}\right)}^{2}}$$ (2)

### CG ABFE calculations

ABFE calculations (*35*) were performed on the CG PTCH1 complex with a cholesterol molecule bound to the TMD SSD (just PTCH1-molA), the ECD SBD, and a midpoint along PMF-1a at approximately 2.4. nm on the reaction coordinate (for PTCH1-molA and PTCH1-molB). Initial poses reflect those used to seed the steered MD simulations as described previously. In all cases, the systems were trimmed to ca. 15 nm by 15 nm by 15 nm, and for the TMD and midpoint systems, all cholesterol apart from the target molecule were removed from the system, followed by 200 ns of equilibration.

ABFE calculations were essentially run as previously described (table S2) (*60*, *64*). Briefly, the target cholesterol is fully decoupled from the system along an alchemical coordinate, λ. The Lennard-Jones interactions of the cholesterol particles were switched off over 29 windows, using a step size of 0.05 for λ = 0 to 0.6 and a step size of 0.25 for λ = 0.6 to 1. A soft-core potential was used, with an α of 0.5 and a σ of 0.3. For each window, the systems were minimized using steepest descents before 3 × 250 ns of production simulations, using a stochastic integrator at 323 K, with pressure held at 1 bar using the Parrinello-Rahman barostat (*65*), with a τ_p_ of 4 ps, and with a compressibility of 5 × 10^−5^ bar^−1^. Throughout the λ windows, flat-bottomed distance restraints of 10 kJ mol^−1^ nm^−2^ were applied between the cholesterol and its bound state to keep it in the binding site at high values of λ (*66*, *67*). The bound state was defined using two dummy beads whose position updated in relation to reference beads in the binding site: E274 + S331 in the SBD site for both states, A936 + Y1013/Y801 in the midpoint site for PTCH1-molA/B, and A478 + V481 for the SSD pose. At low values of λ, the binding site itself restricts the cholesterol molecule, and the restraints have a negligible effect. At high values of λ, the restraints have a larger impact on the dynamics of the cholesterol. These are accounted for using a gas phase λ coordinate whereby the restraints on the cholesterol were switched from a 0.8-nm COM distance restraint to the described flat-bottomed distance restraints in steps of 0, 0.05, 0.10, 0.20, 0.50, and 1.00. For each window, 3 × 250 ns of simulations were run. This coordinate was used to complete the thermodynamic cycle, as described in (*60*). The computed energies were constructed into energy landscapes along λ using the multistate Bennett acceptance ratio (MBAR) (*68*) as implemented in alchemical analysis (*69*).

### Atomistic simulations of PTCH1

The cryo-EM structure of PTCH1 with a putative ion density visible in the TMD (PDB: 6DMY) (*5*) was selected for use in atomistic simulations (Fig. 1A). PTCH1 was set up and embedded within a POPC:CHOL (3:1) bilayer as described in the Supplementary Materials. A Na^+^ ion was positioned within the TMD according to the cryo-EM density (*5*, *15*), and the system was solvated with TIP4P water and approximately 0.15 M NaCl. Two rounds of steepest decent energy minimization were proceeded by 2 × 5 ns of constant number, velocity and temperature (NVT) and constant number, pressure and temperature (NPT) equilibration steps with restraints applied to PTCH1.

Atomistic simulations (3 × 100 ns) were performed with one Na^+^ initially bound in the TMD. Additional simulations (3 × 100 ns) were run in the presence of −100 and −200 mV membrane voltages. Simulations (3 × 50 ns) were also run without Na^+^ bound, and simulations (3 × 100 ns) were performed in the presence of 0.15 KCl in replace of NaCl. Simulations (3 × 100 ns) were initiated with either 3× Na^+^ or 3× K^+^ ions bound at equivalent positions to in the DISP1 structure (PDB: 7RPH) (*46*). In silico mutants of PTCH1 were each simulated for 3 × 50 ns (Supplementary Materials and table S3).

GROMACS 2018 and 2019 (www.gromacs.org) were used to run simulations (*61*). A 2-fs time step was used, and the CHARMM36 force field (*70*) described all components. The Nosé-Hoover thermostat was used to maintain temperature at 310 K (τ_t_ = 0.5 ps) (*71*, *72*). The Parrinello-Rahman barostat (*65*) was used to maintain semi-isotropic pressure at 1 bar (τ_p_ = 2.0 ps), with a compressibility of 4.5 × 10^−5^ bar^−1^. The Particle-Mesh-Ewald (PME) method was used to model long-range electrostatic interactions (*73*). van der Waals (VDW) interactions were cut off at 1.2 nm. Bond lengths were constrained to the equilibrium values using the LINCS algorithm (*74*). A dispersion correction was not applied.

### Atomistic simulations of DISP1

A DISP1 structure bound to three Na^+^ ions was used in atomistic simulations (PDB: 7RPH; “R conformation”) (*46*). DISP1 was set up and simulated as described in the Supplementary Materials in distinct ion-bound or apo states, three of which were generated by sequential ion removal from the final frame of the previous trajectory (3 × 100 ns × 5 ion-bound states) (table S3). Simulation parameters were identical to those described previously for PTCH1.

### Cation FEP

The end snapshot from one 100-ns atomistic simulations of PTCH1 with Na^+^ bound in the TMD was used in FEP calculations (*69*). Na^+^ was alchemically changed to K^+^ over 31 windows by independently modifying the mass (λ windows 0 to 10) and VDW radii (λ windows 11 to 30) of the bound ion using a dual topology approach. Mass modification did not contribute substantially to the overall free energy, and therefore, a λ step size of 0.1 was used in windows 0 to 10 and a step size of 0.05 in windows 11 to 30. A soft-core parameter (σ = 0.3) was used for Lennard-Jones interactions. Each window was simulated for 50 ns using the protocol described previously for atomistic simulations of PTCH1 with the exception that a stochastic dynamics integrator was used. Perturbation of Na^+^ to K^+^ in free solution was achieved using a single Na^+^ in a 3-nm by 3-nm by 3-nm TIP4P water box. A single Cl^−^ counterion was present. The solvent box was equilibrated for 20 ns before use in FEP calculations to allow water to equilibrate around the ion. Each window was simulated for 5 ns using the same protocol described for the PTCH1-bound Na^+^ systems. Five repeats of each FEP calculation were performed. Five repeats of the inverse perturbations (K^+^ to Na^+^) in the bound and free states were also performed, which gave free energy values of the same magnitude but inverse signs (fig. S7). Alchemical analysis (*69*) was used in calculation of the overall free energy values from λ windows. There was good agreement between analysis methods, and so, values calculated using MBAR (*68*) are reported as the free energy mean and SD between repeats (fig. S10). The first 200 ps of each window were discarded to allow for equilibration.

### Generation of stable cell lines expressing PTCH1 mutants

#### PTCH1 construct generation

hPtch1, comprising amino acids 75 to 1185 including a deletion of intracellular loop 3 (Δ630 to 717), was cloned into the pHR-CMV-TetO2 vector (Addgene plasmid #113893) (*75*). For functional studies, the following single- or double-point mutations were introduced: V510F, D513Y, L517C/P1125C, L570C/V1081C, and P115R/N802E.

#### Virus generation

Viral supernatant containing the hPtch1 variants was generated using human embryonic kidney (HEK) 293T cells (American Type Culture Collection, catalog no. CRL03216) and then added to *Ptch1* knockout mouse embryonic fibroblasts (*Ptch1*^−/−^ MEFs) (*37*). To generate virus, 2.5 × 10^6^ HEK 293T cells were plated on 6-cm plates in 3 ml of antibiotic-free high-glucose Dulbecco’s modified Eagle’s medium (DMEM) supplemented with l-glutamine, sodium pyruvate, and nonessential amino acids. Twenty-four hours later, cells were transfected by mixing 500 μl of room temperature Opti-MEM, 5 μg of the pHR-CMV-TetO2 plasmid containing the hPtch1 gene, 2.5 μg of pVSVG, 3.75 μg of psPAX2, and 45 μl of polyethylenimine (PEI) (4 μl of PEI to 1 μg of DNA). After incubating this mixture at room temperature for 20 min, it was added dropwise to the 6-cm dish of HEK 293T cells. The following day, the medium on the 293T plates was exchanged for a fresh medium. At this time, 6-cm plates were seeded with 2 × 10^5^ *Ptch1*^−/−^ MEFs. Twenty-four hours later, the virus-containing medium was harvested from the 293T cells, and polybrene was added to 4 μg/ml. This undiluted viral supernatant was added directly to the *Ptch1*^−/−^ MEFs. Fresh antibiotic-free DMEM was added to the 293T cells for a second round of virus production, followed by a second infection of the *Ptch1*^−/−^ MEFs 24 hours later.

#### Isolation of Ptch1-expressing cells and validation

*Ptch1*^−/−^ MEFS containing the integrated hPtch1 variants were trypsinized and analyzed compared to an uninfected (control) population using fluorescence activated cell sorting. A population of cells with fluorescence from the integrated hPtch1-mVenus-1D4 construct was clearly visible. This population was used to set a sorting gate for the cells containing the hPtch1 gene. Approximately 250,000 cells were sorted and then plated into a 6-cm dish. Once these cells were grown to a sufficient density, they were seeded into additional plates for Western blot analysis (*41*) to confirm expression of the hPTCH1-mVenus-1D4 gene. An anti–green fluorescent protein antibody (Novus Biologicals, catalog no. NB600-308, RRID: AB_10003058) was used to detect PTCH1 expression levels, and anti-P38 (Abcam, catalog no. ab7952, RRID: AB_306166) was used as a gel loading control.

#### Statistical analysis

Figures 2E and 5D were generated and analyzed in GraphPad Prism 9. Figure S5 was made in Adobe Illustrator CS6. All statistical analyses comparing two datasets used a Student’s *t* test with Welch’s correction. All comparisons shown were prespecified. Throughout the paper, the *P* values for the comparisons from GraphPad Prism 9 are denoted on the graphs according to the following keys: not significant (ns) *P* > 0.05, *P ≤ 0.05, **P ≤ 0.01, ***P ≤ 0.001, and ****P ≤ 0.0001. Unless indicated otherwise, all experiments were performed three times independently with similar results. Exact *P* values are provided within the figure legends.

## Supplementary Materials

### This PDF file includes:

Figs. S1 to S7 Tables S1 to S4 References

### Other Supplementary Material for this manuscript includes the following:

PTCH1 construct sequences
