# Supplementary material for: The energetics and ion coupling of cholesterol transport through Patched1
Source: Sci Adv. 2023 Aug 23;9(34):eadh1609. doi: 10.1126/sciadv.adh1609 (PMC10446486; doi:10.1126/sciadv.adh1609)
Supplement: Supplementary file 1 — Figs. S1 to S7 Tables S1 to S4 References [file sciadv.adh1609_sm.pdf]

Science Advances
AAAS

Supplementary Materials for

The energetics and ion coupling of cholesterol transport through Patched1

T. Bertie Ansell *et al.*

Corresponding author: Mark S. P. Sansom, mark.sansom@bioch.ox.ac.uk

Sci. Adv. **9**, eadh1609 (2023)
DOI: 10.1126/sciadv.adh1609

The PDF file includes:

Figs. S1 to S10
Tables S1 to S4
References

Other Supplementary Material for this manuscript includes the following:

PTCH1 construct sequences

Extended Methods:

Setup of coarse-grained potential of mean force calculations

The following parameters were used in all CG simulations unless otherwise stated. The MARTINI 2.2 forcefield was used to describe all components[32,76]. Protein coarse-graining was achieved using the *martinize.py* script with the ElNeDyn elastic network, a spring force constant of 500 kJ mol^{-1} nm^{-2} and a cut-off of 0.9 nm [77]. Membranes were built using *insane.py*[78]. All systems were solvated using MARTINI water and approximately 0.15 M NaCl. A velocity (V-)rescaling thermostat[79] was used to maintain temperature at 310 K (τ_t=1.0 ps). The Parrinello-Rahman barostat[65] was used to maintain pressure at 1 bar (τ_p=12.0 ps) with a compressibility of $3x10^{-4}$ bar^{-1}. The timestep was 20 fs.

PMF-1: between the SSD and SBD and PMF-2: between the SSD and bulk membrane

The SSD of PTCH1-molA and PTCH1-molB (PDB: 6RVD[15]) were aligned to the x axis to assist generation of the reaction coordinate and the surrounding membrane and solvent expanded to a box size of 20 x 20 x 19 nm^3 using gmx genconf and VMD[80]. Two round of steepest decent energy minimisation were performed to relax the equilibrated bilayer around PTCH1. For PMF-1a the 'SHH-cholesterol' or 'free cholesterol' molecules were positioned in PTCH1-molA and PTCH1-molB ECDs respectively according to the cryo-EM densities[15]. PMF-1b was initiated with cholesterol bound at the base of the ECD, continuing from the final window in PMF-1a from PTCH1-molB. Since the 'free cholesterol' bound within PTCH1-molB was positioned with the ROH bead orientated towards the bilayer, a bash script was used to invert the cholesterol orientation in frames generated from the steered MD simulations to generate the PTCH1 'SHH-cholesterol' conformations. This was due to difficulties obtaining a reasonable steered MD simulation initiated from the final window of PMF-1a from PTCH1-molA and to standardise the reaction coordinate. For PMF-2 cholesterol was not present in the PTCH1 ECD. Instead, snapshots were selected with cholesterol bound to the SSD with either the ROH bead (equivalent to the β_3-OH group) pointing towards the extracellular leaflet headgroups ('OH-up') for both the 'SHH-cholesterol' (PTCH1-molA) and 'free cholesterol' (PTCH1-molB) orientations, or towards the bilayer midplane ('OH-down').

PMF-3: between the bulk membrane and solvent

A 10 x 10 x 18 nm^3 POPC:CHOL (3:1) bilayer patch without protein was built using *insane.py*[59,78]. The bilayer was solvated using MARTINI water[32] and 0.15 M NaCl before two round of steepest decent energy minimisation. A cholesterol molecule at the centre of the bilayer was selected for use in PMF calculations.

PMF-4: between the SBD and solvent

The ECD of PTCH1-molA and PTCH1-molB (residues A119–D436 and R772–G1023) from the PTCH1-SHH (2:1) structure (PDB: 6RVD[15]) was extracted and coarse-grained[32]. The 'SHH-cholesterol' and 'free cholesterol' molecules were positioned in the SBD of PTCH1-molA and PTCH1-molB respectively accordioning to their positions in the cryo-EM density[15] (and identically to in PMF-1a). The PTCH1 ECDs were positioned at one end of a 9 x 9 x 18 nm^3 box with the SBD aligned to the z axis. The system was solvated with MARTINI water[32] and approximately 0.15 M NaCl followed by two rounds of steepest decent energy minimisation.

Execution and analysis of coarse-grained potential of mean force calculations

Steered MD simulations were used to generate 1D reaction coordinates for each path of the PTCH1-molA and PTCH1-molB free energy cycles. The 1D reaction coordinate was generated by application of a distance dependant pulling force along specified axes between the COM of the cholesterol and the following selections: PMF-1a (I1092 BB bead), PMF-1b (F800 BB bead), PMF-2 (P504 BB bead), PMF-3 (COM bilayer) and PMF-4 (S331 BB bead). The umbrella pulling force was 1000 kJ mol^{-1} nm^{-2} and pulling rates of 0.1 nm ns^{-1} (PMF-1a, 2, 3, 4) or 10 nm ns^{-1} (PMF-1b) were used. Position restraints of 1000 kJ mol^{-1} nm^{-2} were applied to the following backbone beads to prevent protein rotation: PMF-1a/b, (A1088, A1157), PMF-2 (A1088, A1157) and PMF-4 (A182, A239). Frames were taken with spacing 0.05 nm along the reaction coordinate and subjected to 1-3 µs of simulation. For each window an umbrella pulling force of 1000 kJ mol^{-1} nm^{-2} was used to confine the cholesterol position along the reaction coordinate. Further details of the number of windows, simulations times and PMF convergence are given in Table S1 and Fig. S9. PMF profile analysis was assisted using the *pmf.py* tool[60] and the weighted-histogram analysis method[62] implemented in GROMACS (bootstrapped 2000 times).

Setup for atomistic simulations of PTCH1

A PTCH1 structure with ion-like density within the TMD (PDB: 6DMY)[5] was used in atomistic simulations. SHH, ligands and metal ions were removed. A short linker was modelled between TM6 and TM7 using Modeller 9.20[81] to give an overall sequence of (TM6)DRR-LDIFCC//TKWTLSSFAE-KHY(TM7), in accordance with the linker used in CG simulations of PTCH1 (PDB: 6RVD[15]). A Na^+ ion was positioned according to the density in the centre of the TMD[5]. The H++ server and propKa[82,83] were used to predict the pKa of titratable groups (Table S4), revealing at least two residues of the anionic triad to be protonated at pH 7 in the absence of bound Na^+, decreasing to one protonated residue when Na^+ was present. The central residue, E1095, was therefore protonated using the CHARMM-GUI PDB generator which was also used to rename atoms to be compatible with the CHARMM-36 forcefield and model disulphide bonds between C203-C226, C234-C327, and C296-C304[70,84]. PTCH1 was embedded in a symmetric POPC:CHOL (3:1) membrane generated using the CHARMM-GUI bilayer

builder[85,86] and solvated using TIP4P water[87] and approximately 0.15 M NaCl. PTCH1 was subsequently energy minimised and equilibrated in 2 x 5 ns NVT and NPT steps with restraints applied to the PTCH1 backbone.

Application of a membrane potential

Atomistic simulations of the above setup were also run in the presence of a -100 mV or -200 mV membrane voltage ($\Delta V = E.L_z$, E=-7.1429 or –14.286 mV/nm, L_z=14 nm) for 3 x 100 ns each. Voltage is reported as inside relative to outside and was achieved using the constant electric field method[88].

In silico mutation screen

Modeller 9.20 was used to induce single amino acid substitutions at a specified location and induce disulphide bond formation in the cross-linked mutants[81]. The following mutations were performed: V510G, V510A, V510T, V510F, I1092A, L517C-P1125C, H1099C-S547C. Atomistic simulations of WT PTCH1 and each PTCH1 mutant were performed as described above with the exception that Na^+ was not initially bound in the TMD and each system was simulated for 3 x 50 ns.

Setup for atomistic simulations of DISP1

The DISP1 structure with three Na^+ ions bound was used in simulations (PDB: 7RPH, 'R conformation')[46]. Detergent ligands were removed, and the missing loop modelled using Modeller9.20[81]. CHARMM-GUI was used to generate DISP1 parameters (separated into two chains to account for the Furin cleavage site) and the anionic triad residue D1049 was protonated for consistency with the PTCH1 simulations[70,84]. DISP1 was simulated in the '3x Na^+' bound and apo states for 3 x 100 ns each. The '2x Na^+' state was generated from the end snapshots of the '3x Na^+' state by removing the ion missing in an alternative 'T conformation' of DISP1 (PDB: 7RPI)[46] bound to two Na^+ ions. The '1x Na^+' and '0x Na^+' states were generated by an identical process of sequential ion removal from the end snapshots of previous simulations. The ion retained in the '1x Na^+' state was chosen based on the apo state simulations whereby an ion spontaneously bound to D572 in all replicates. Each subsequent ion state was also simulated for 3 x 100 ns.

Analysis

Protein-lipid interactions

The PyLipID analysis toolkit[89] was used to identify residues in the PTCH1 ECD which interacted with ECD bound cholesterol (https://github.com/wlsong/PyLipID). All windows in the PMF-1a profile or windows surrounding energetic bottlenecks in the PMF-1a profile were analysed collectively using PyLipID with a single 0.6 nm interaction cut-off. The top four residues with the highest occupancy i.e. those residues within 0.6 nm of cholesterol for the largest fraction of the window simulation times were reported for each bottleneck.

Water within PTCH1 and DISP1

MDAnalysis[90] was used to analyse water within PTCH1 TMD. For each timepoint in the trajectory the O atom of water within a cylinder (radius 1.3 nm in xy) centred around the Cα atoms of residues V510 and I1092 was considered to localise within the TMD. For analysis of water z coordinates with time the length of the cylinder was 4 nm in z whereas for average water densities in the mutant screen a 3 nm cylinder was used to reduce contributions from bulk water molecules. Water within the extracellular and intracellular halves of the TMD was defined as having z coordinate of the O atom localised within the cylinder and either above or below the z coordinate of the midpoint between the Cα atoms V510 and I1092. Only the final 10 ns of each 50 ns simulation of PTCH1 were used in comparison of TMD water density to allow water to equilibrate in the TMD. PyMol and VMD were used to visualise trajectories[80].

Analysis of water within the intracellular TMD half of DISP1 was performed identically to described above with the cylinder midpoint defined using the Cα atoms of I568 and L1046.

<u>PTCH1 construct sequences</u>

Details of PTCH1 constructs are provided within the accompanying Supplementary excel file.

Supplementary Figures

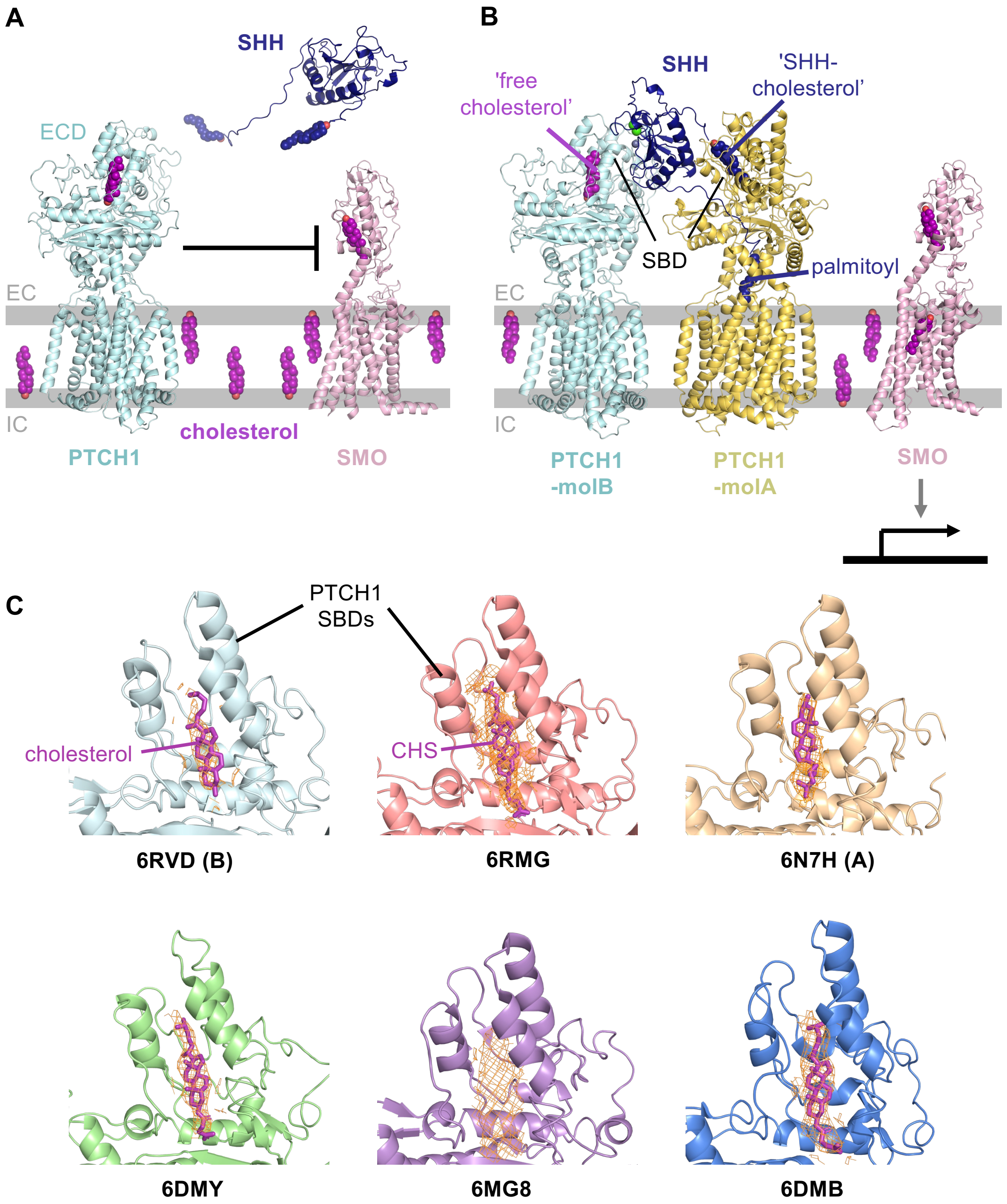

Figure S1: Patched1 and the vertebrate Hedgehog signalling pathway. A) Patched1 (PTCH1, light blue) inhibits Smoothened (SMO, pink) in the absence of Sonic Hedgehog (SHH, dark blue). **B)** SHH binds to PTCH1 via a metal binding interface (PTCH1-molB) or insertion of terminal cholesteryl/palmitoyl attachments (PTCH1-molA, yellow), the later relieves SMO inhibition and initiates Hedgehog (HH) signalling. Proteins are shown in cartoon representation and cholesterol/palmitate are shown as spheres. Extracellular (EC) and intracellular (IC) leaflets are indicated. **C)** Cholesterol and cholesterol-hemisuccinate densities (orange) within the sterol binding domain (SBD) of existing PTCH1 structures. Modelled sterols are shown in purple.

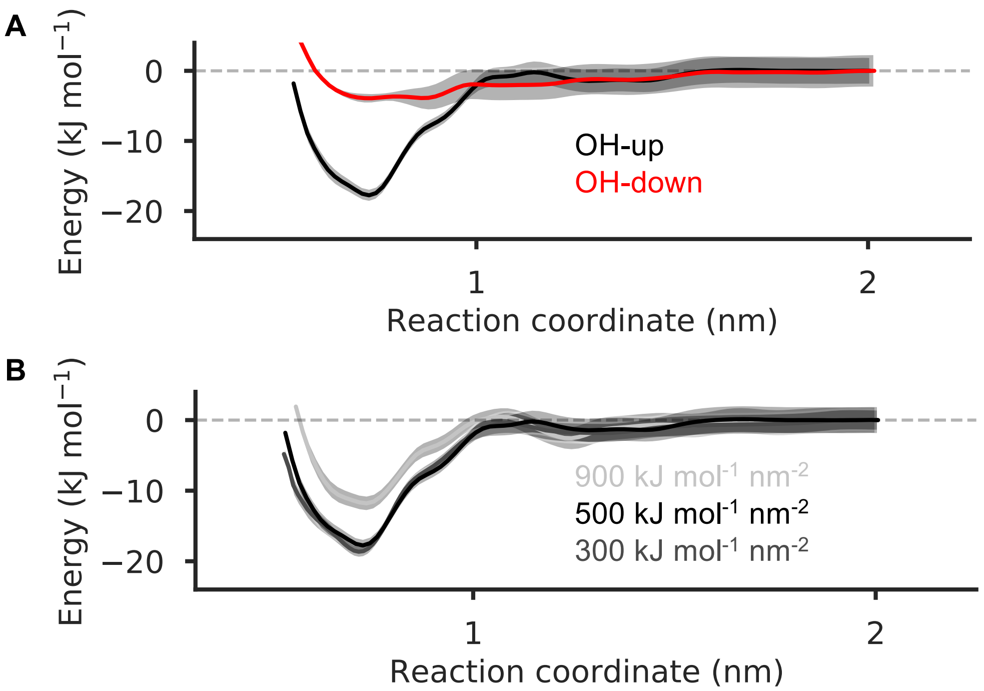

Figure S2: Cholesterol binds the PTCH1 SSD in the OH-up orientation.
A) Coarse-grained (CG) potential of mean force (PMF) profiles for movement of cholesterol away from the PTCH1 sterol sensing domain (SSD) when bound with the ROH bead (equivalent to the β3-hydroxyl group) facing towards the lipid phosphate groups (OH-up, black) or towards the bilayer midplane (OH-down, red). **B)** As in **A** for the OH-up conformation whereby the CG ElNeDyn elastic network force-constant was modified away from the default used throughout this study of 500 kJ mol^{-1} nm^{-2} (black) to 300 kJ mol^{-1} nm^{-2} (grey) or 900 kJ mol^{-1} nm^{-2} (light grey). Bayesian bootstrapping (2000 rounds) was used to estimate profile errors (grey).

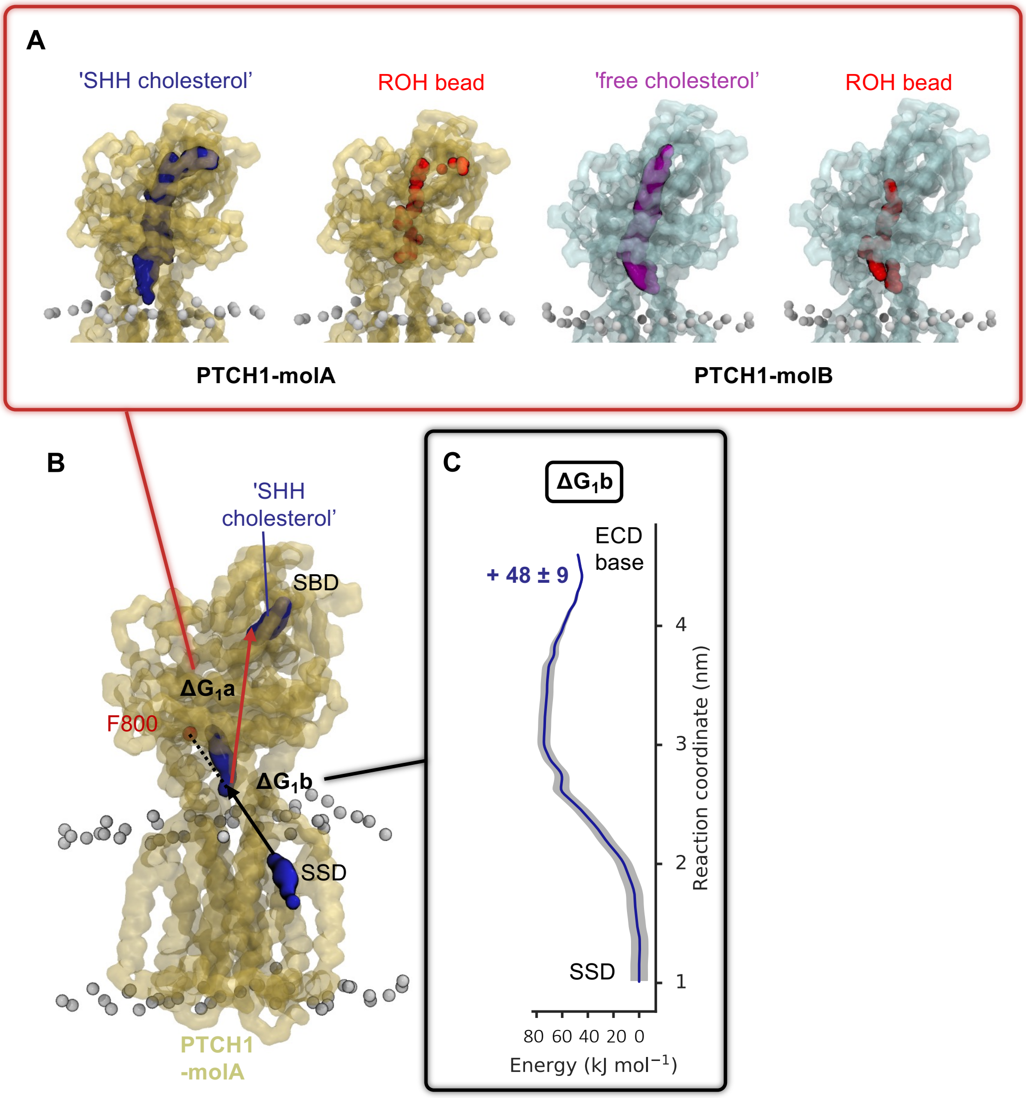

Figure S3: Cholesterol pathways through the PTCH1 ECD in PMF calculations. A) Coarse-grained (CG) representation of PTCH1-molA (yellow) and PTCH1-molB (blue) overlayed with the position of the 'SHH cholesterol' (dark blue) and 'free cholesterol' molecules, or their associated ROH beads (red) across all PMF-1a windows. The free energy profile for ΔG_1a is shown in Fig. 2D. **B)** Snapshots indicating movement of cholesterol between the sterol sensing domain (SSD) and the base of PTCH1 ECD in PMF-1b (residues used in the steered MD are labelled in red) in CG PTCH1-molA (yellow). **C)** PMF profile for cholesterol movement between the SSD and ECD base (ΔG_1b). Bootstrapping errors (2000 rounds) are shown in grey. This PMF was calculated using the 'SHH-cholesterol' orientation bound PTCH1-molA.

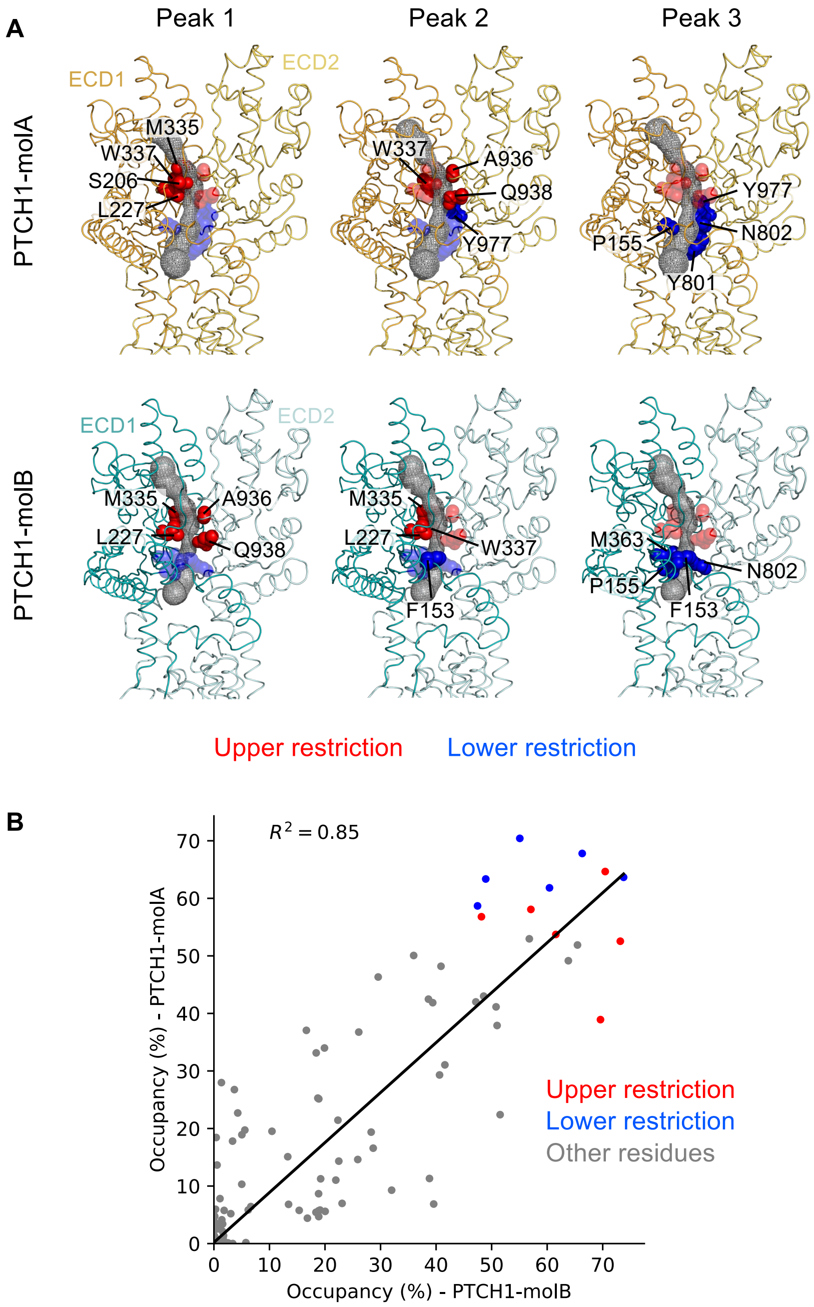

Figure S4: Energetic restrictions within PTCH1 sterol transport tunnels.
A) Conserved energetic peaks within the ECD (numbered 1-3 in Fig. 2D) of PTCH1-molA (yellow) and PTCH1-molB (light blue). The four residues with highest cholesterol interaction occupancy at each peak were identified from umbrella sampling windows using PyLipID[89] and a 0.6 nm cut-off. Residues are shown as spheres coloured by localisation within the upper (red) or lower (blue) restrictions in the PTCH1 ECD, surrounding putative ECD sterol transport tunnels (grey mesh). Residues corresponding to a particular peak are opaque and labelled, overlaid with those residues which comprise the remaining two peaks (transparent). **B)** Cholesterol interaction occupancy of equivalent residues in PTCH1-molA and PTCH1-molB across PMF-1a windows, indicating the same high occupancy residues contribute to formation of the upper (red) and lower (blue) restrictions between PTCH1 conformations. Cholesterol contacts were defined using a 0.6 nm cut-off.

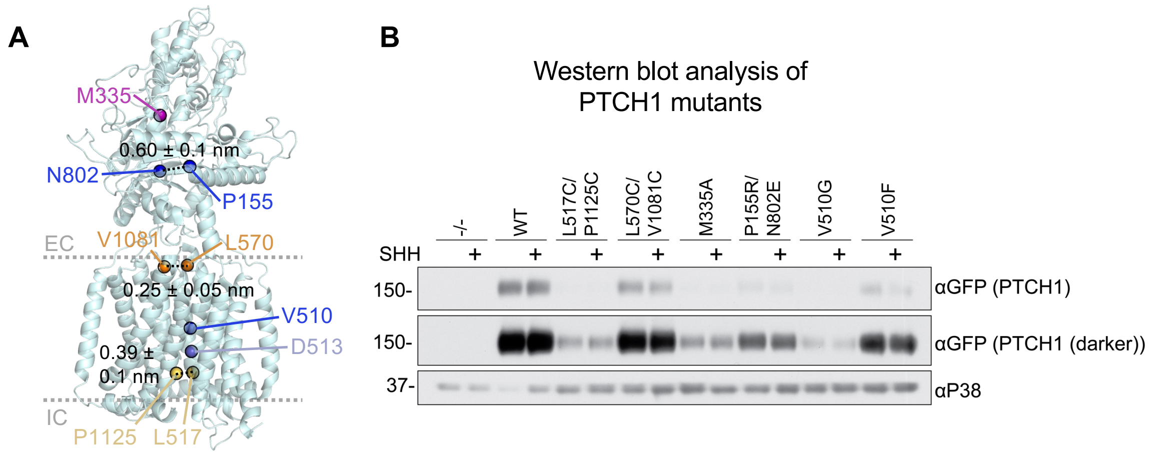

Figure S5: Expression levels of PTCH1 mutants.
A) Location of PTCH1 mutants mapped onto the PTCH1 structure (PDB: 6DMY). The minimum distance between labelled residues across simulations of WT PTCH1 is indicated. These distances were used to inform PTCH1 mutant design. **B)** Western blot analysis of PTCH1 WT and mutant variant expression levels in *Ptch1*$^{-/-}$ mouse embryonic fibroblasts in the presence or absence of SHH addition.

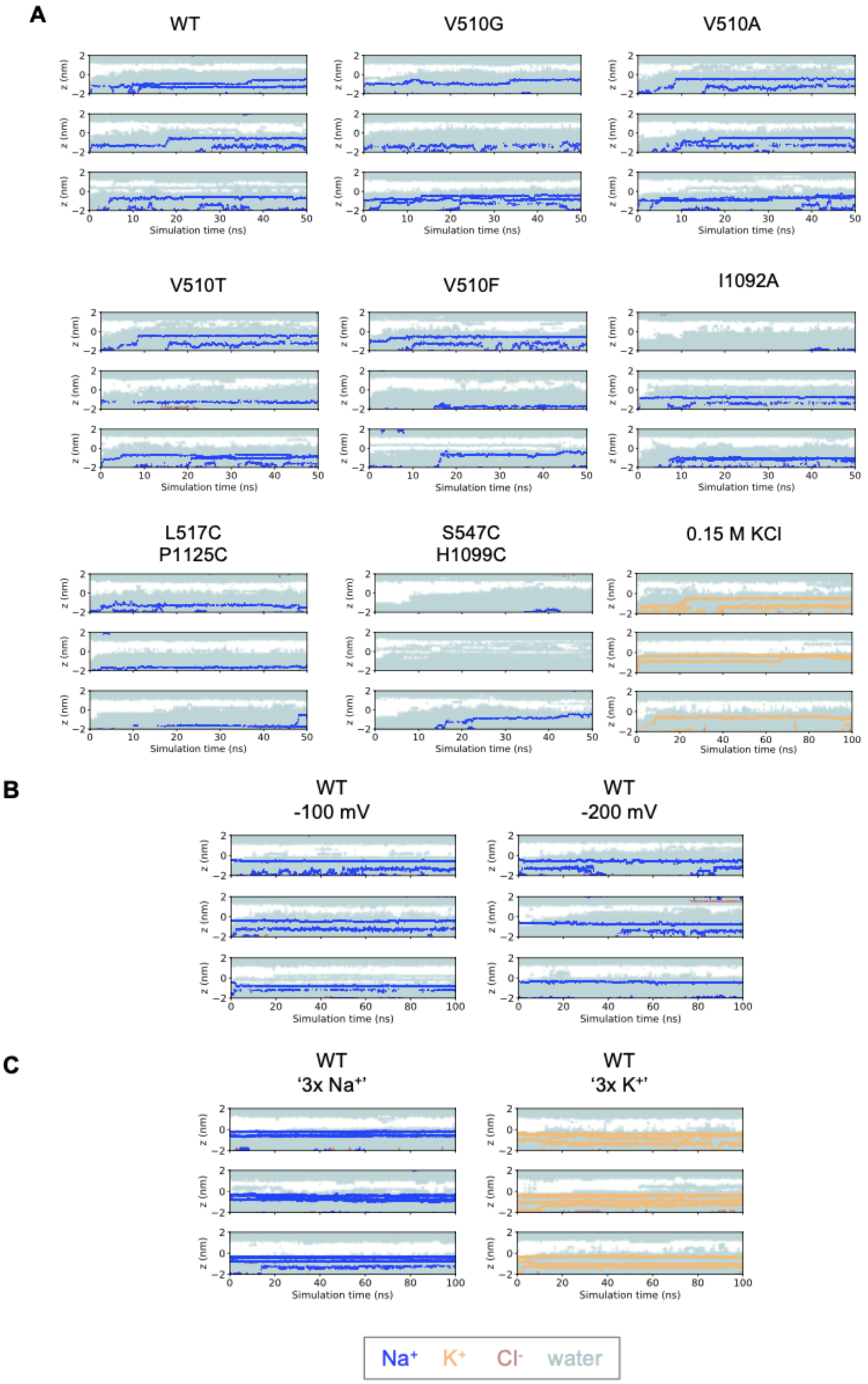

A
WT
V510G
V510A
V510T
V510F
I1092A
L517C
P1125C
S547C
H1099C
0.15 M KCl
B
WT
-100 mV
WT
-200 mV
C
WT
'3x Na+'
WT
'3x K+'
z (nm)
Simulation time (ns)
Na+
K+
Cl-
water

Figure S6: Water and ions within the TMD of PTCH1 mutants.

A) The z coordinates of water oxygen atoms (light blue), Na^+ (blue), K^+ (yellow) and Cl^- (salmon) ions localized within the PTCH1 TMD. A cylinder (length 4 nm, radius 1.3 nm) centred on the midpoint of V520 and I1092 Cα atoms was to identify water and ions within the PTCH1 TMD for WT and labelled PTCH1 mutants (V510G, V510A, V5105, V510F, I1092A, L517C/P1125C, S547C/H1099C). Simulations were initiated without Na^+/K^+ bound within the TMD and simulated for 3 x 50 ns or 3 x 100 ns replicates. **B)** Water and ions within the WT PTCH1 TMD (defined as in **A**) accounting in the presence of a -100 mV or -200 mV membrane potential (see methods). **C)** As in **A-B** for WT PTCH1 simulations initiated with 3 x Na^+ or 3 x K^+ ions bound at equivalent positions to three Na^+ ions observed within a structure of DISP1 (PDB: 7RPH).

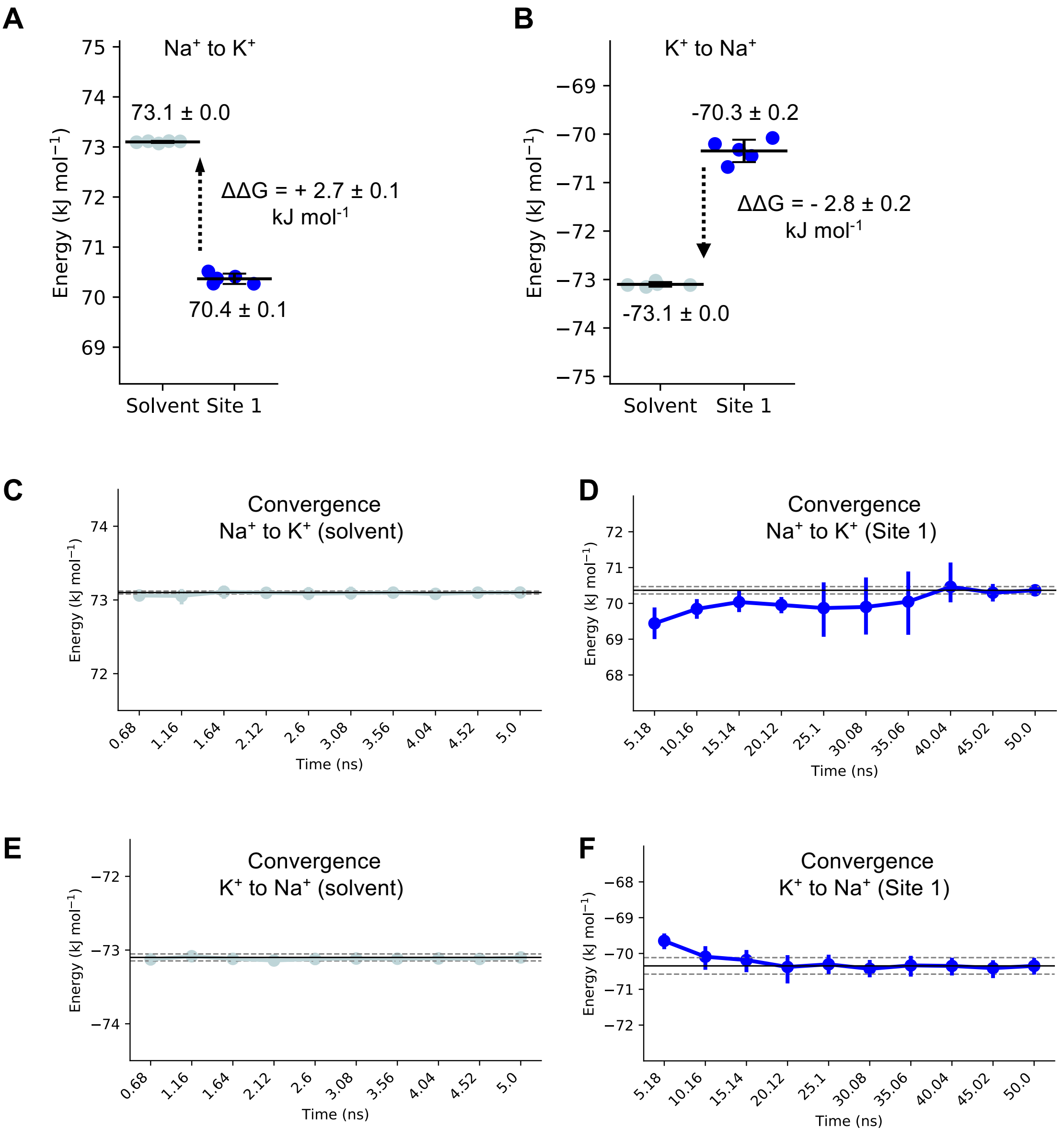

Figure S7: Convergence of FEP calculations.

Free energy perturbation of **A)** Na^+ to K^+ or **B)** K^+ to Na^+ when bound to PTCH1 Site 1 or in solvent. The forward and reverse perturbations are in agreement. **C-F)** Convergence of FEP calculations as a fraction of λ window length for **C)** Na^+ to K^+ in solvent, **D)** Na^+ to K^+ at PTCH1 Site 1 **E)** K^+ to Na^+ in solvent and **F)** K^+ to Na^+ at PTCH1 Site 1. Error bars indicate the minimum/maximum values (**A/B**) or standard deviation between 5 FEP replicates (**C-F**).

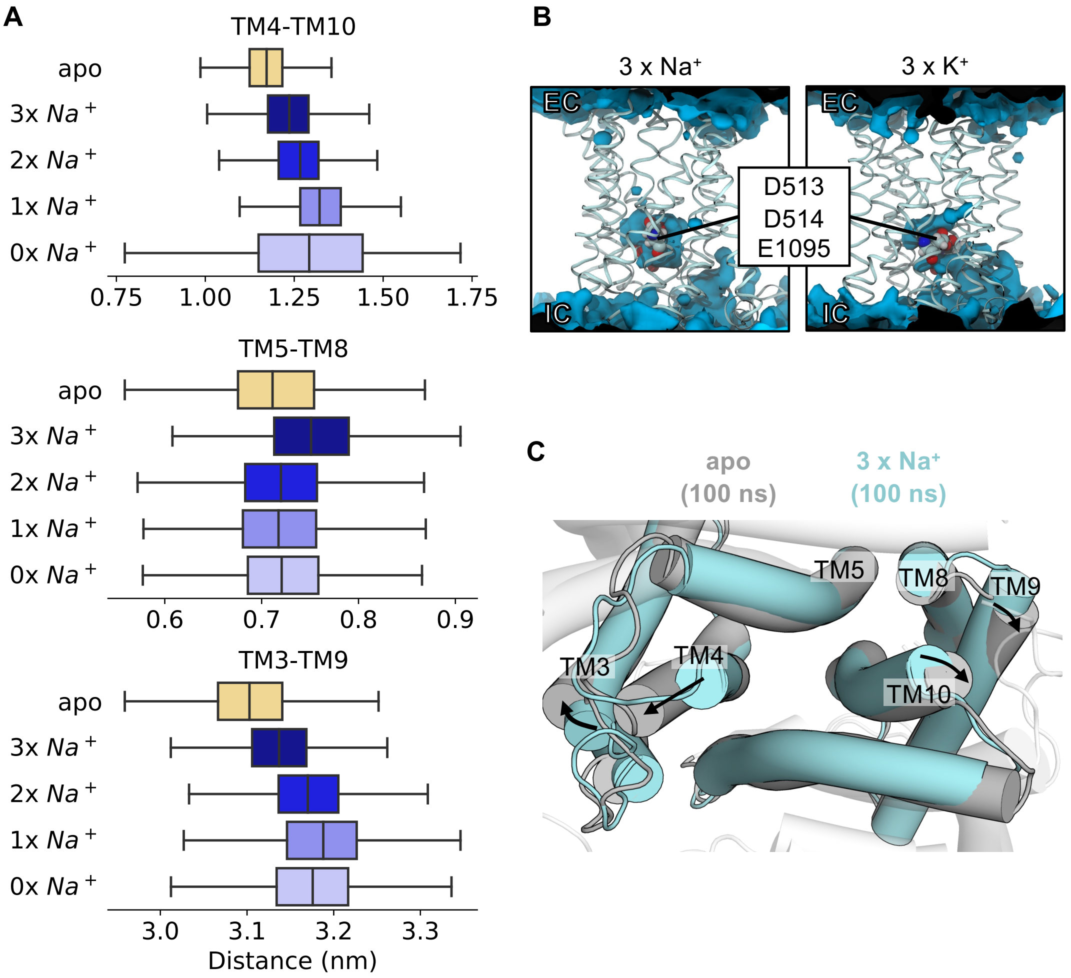

Figure S8: DISP1 extracellular TMD distances and PTCH1 TMD conformational changes.

A) Minimum distances between the extracellular portions of transmembrane helices across 3 x 100 ns simulations of DISP1 in distinct ion coupled states. Distances were defined between the Cα atoms of A561-T1039 (TM4-TM10), V623-A985 (TM5-TM8) and Y542-V1022 (TM3-TM9). **B)** Time averaged water density (blue isosurface) across 100 ns simulations of PTCH1 (PDB: 6DMY) initiated with either 3 x Na^+ or 3 x K^+ ions bound within the TMD. Anionic triad residues are shown as spheres. **C)** Comparison of the intracellular PTCH1 TMD helices at the end of 100 ns atomistic simulations initiated in either an apo conformation or with 3x Na^+ ions bound within the TMD.

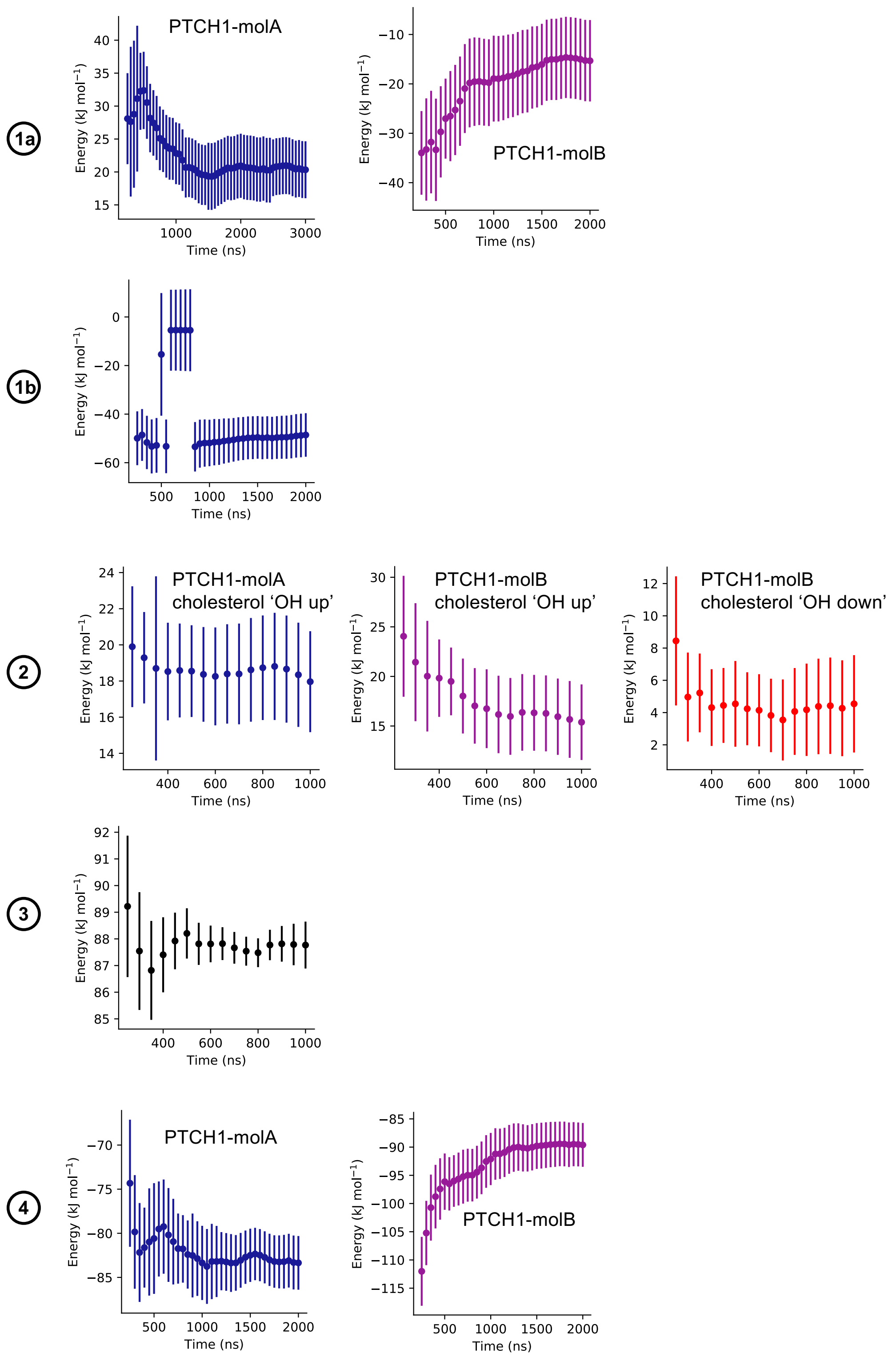

Figure S9: Convergence of PMF calculations.
Free energy from PMF calculations as a fraction of the per window simulation time analysed. Free energy values were calculated using gmx wham with 200 Bayesian Bootstraps, discarding the first 200 ns of each window as equilibration time. PMFs are numbered according to Fig. 1-2 and coloured according to 'SHH-cholesterol' (blue, bound to PTCH1-molA) and 'free cholesterol' (purple, bound to PTCH1-molB) orientations.

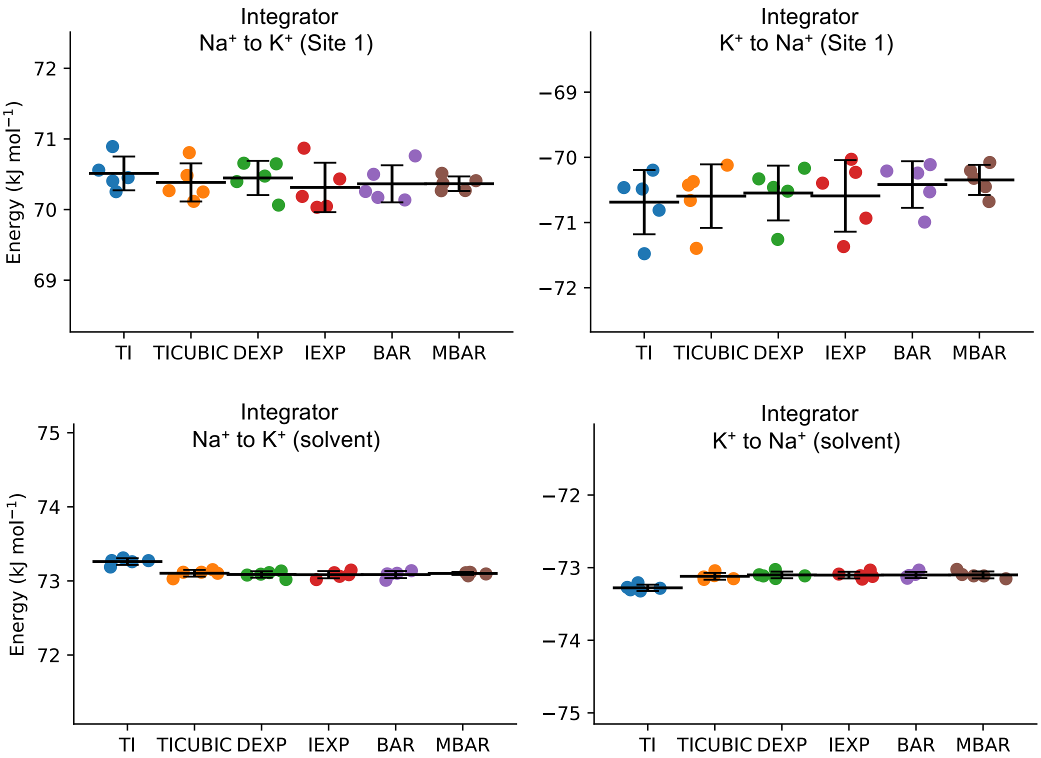

Figure S10: Choice of integrator in FEP calculations.
FEP values ion perturbations predicted using different integrators. See Fig. S7 legend for FEP details. The MBAR integrator was used to report FEP values.

Supplementary Tables

Table S1: Summary of the number of windows, simulations time per window and free energy value obtained from PTCH1 CG PMF calculations. The number of windows correspond to those included in the *gmx wham* step.

**PMF**	**System**	**No. windows**	**Window length (µs)**	**Total simulation time (µs)**	**ΔG (kJ mol^{-1})**
1a	PTCH1-molA ‘SHH-cholesterol’	92	3	276	+20 ± 4
	PTCH1-molB ‘free cholesterol’	99	2	198	-15 ± 7
1b	PTCH1-molA ‘SHH-cholesterol’	98	2	196	+48 ± 9
2	SSD Cholesterol ‘OH up’ PTCH1-molA	65	1	65	+18 ± 3
	SSD Cholesterol ‘OH up’ PTCH1-molB	56	1	56	+16± 4
	SSD Cholesterol ‘OH down’ PTCH1-molB	65	1	65	+4 ± 3
3	Membrane cholesterol	76	1	76	+88 ± 1
4	PTCH1-molA ECD ‘SHH-cholesterol’	112	2	224	-83 ± 3
	PTCH1-molB ECD ‘free cholesterol’	101	2	202	-90 ± 4

Table S2: Summary of the replicates, lambda values and free energies obtained from CG ABFE calculations.

System	Replicates	No. λ windows	Window length (µs)	Total simulation time (µs)	ΔG (kJ mol^{-1})
PTCH1-molA ECD 'SHH-cholesterol'	3	29	0.15	13.05	+92 ± 3
PTCH1-molB ECD 'free cholesterol'	3	29	0.15	13.05	+86 ± 1
PTCH1-molA SSD Cholesterol 'OH up'	3	29	0.15	13.05	+105 ± 1
PTCH1-molA ECD 'mid cholesterol'	3	29	0.15	13.05	+66 ± 1
PTCH1-molB ECD 'mid cholesterol'	3	29	0.15	13.05	+61 ± 2

Table S3: Summary of equilibrium atomistic simulations of PTCH1 and DISP1.

System	PDB	Replicates x Time	Initial ligands/conditions
PTCH1	6DMY	3 x 100 ns	Na^+ (Site 1)
PTCH1	6DMY	3 x 50 ns	apo (in 0.15 M NaCl)
PTCH1	6DMY	3 x 100 ns	apo (in 0.15 M KCl)
PTCH1	6DMY	3 x 100 ns	Na^+ (Site 1), -100 mV membrane potential
PTCH1	6DMY	3 x 100 ns	Na^+ (Site 1), -200 mV membrane potential
PTCH1-mutants	6DMY	3 x 50 ns x 7 mutants	apo (in 0.15 M NaCl)
PTCH1	6DMY	3 x 100 ns	'3x Na^+'
PTCH1	6DMY	3 x 100 ns	'3x K^+'
DISP1	7RPH	3 x 100 ns	apo
DISP1	7RPH	3 x 100 ns	'3x Na^+'
DISP1	7RPH	3 x 100 ns	'2x Na^+'
DISP1	7RPH	3 x 100 ns	'1x Na^+'
DISP1	7RPH	3 x 100 ns	'0x Na^+'

Table S4: pKa values of the PTCH1 anionic triad residues as predicted using the propKa and H++ servers.

Residue	Predicted pKa		
	H++	H++ (Na^+ bound)	PropKa
D513	>12.00	>12.00	8.45
D514	6.26	<0.00	8.24
E1095	11.29	0.90	10.95

REFERENCES AND NOTES

1. J. H. Kong, C. Siebold, R. Rohatgi, Biochemical mechanisms of vertebrate hedgehog signaling. *Development* **146**, dev166892 (2019).

2. V. Marigo, R. A. Davey, Y. Zuo, J. M. Cunningham, C. J. Tabin, Biochemical evidence that Patched is the Hedgehog receptor. *Nature* **384**, 176–179 (1996).

3. F. Wu, Y. Zhang, B. Sun, A. P. McMahon, Y. Wang, Hedgehog signaling: From basic biology to cancer therapy. *Cell Chem. Biol.* **24**, 252–280 (2017).

4. S. X. Atwood, K. Y. Sarin, R. J. Whitson, J. R. Li, G. Kim, M. Rezaee, M. S. Ally, J. Kim, C. Yao, A. L. S. Chang, A. E. Oro, J. Y. Tang, Smoothened variants explain the majority of drug resistance in basal cell carcinoma. *Cancer Cell* **27**, 342–353 (2015).

5. X. Gong, H. Qian, P. Cao, X. Zhao, Q. Zhou, J. Lei, N. Yan, Structural basis for the recognition of Sonic Hedgehog by human Patched1. *Science* **361**, eaas8935 (2018).

6. X. Qi, P. Schmiege, E. Coutavas, J. Wang, X. Li, Structures of human Patched and its complex with native palmitoylated sonic hedgehog. *Nature* **560**, 128–132 (2018).

7. X. Qi, P. Schmiege, E. Coutavas, X. Li, Two Patched molecules engage distinct sites on Hedgehog yielding a signaling-competent complex. *Science* **362**, eaas8843 (2018).

8. C. Qi, G. D. Minin, I. Vercellino, A. Wutz, V. M. Korkhov, Structural basis of sterol recognition by human hedgehog receptor PTCH1. *Sci. Adv.* **5**, eaaw6490 (2019).

9. Y. Zhang, D. P. Bulkley, Y. Xin, K. J. Roberts, D. E. Asarnow, A. Sharma, B. R. Myers, W. Cho, Y. Cheng, P. A. Beachy, Structural basis for cholesterol transport-like activity of the Hedgehog receptor Patched. *Cell* **175**, 1352–1364.e14 (2018).

10. H. Qian, P. Cao, M. Hu, S. Gao, N. Yan, X. Gong, Inhibition of tetrameric Patched1 by Sonic Hedgehog through an asymmetric paradigm. *Nat. Commun.* **10**, 2320 (2019).

11. Y. Zhang, W.-J. Lu, D. P. Bulkley, J. Liang, A. Ralko, S. Han, K. J. Roberts, A. Li, W. Cho, Y. Cheng, A. Manglik, P. A. Beachy, Hedgehog pathway activation through nanobody-mediated conformational blockade of the Patched sterol conduit. *Proc. Natl. Acad. Sci. U.S.A.* **117**, 28838–28846 (2020).

12. P. Huang, B. M. Wierbowski, T. Lian, C. Chan, S. García-Linares, J. Jiang, A. Salic, Structural basis for catalyzed assembly of the Sonic hedgehog–Patched1 signaling complex. *Dev. Cell* **57**, 670–685.e8 (2022).

13. Y. Luo, G. Wan, X. Zhang, X. Zhou, Q. Wang, J. Fan, H. Cai, L. Ma, H. Wu, Q. Qu, Y. Cong, Y. Zhao, D. Li, Cryo-EM study of Patched in lipid nanodisc suggests a structural basis for its clustering in caveolae. *Structure* **29**, 1286–1294.e6 (2021).

14. A. Radhakrishnan, R. Rohatgi, C. Siebold, Cholesterol access in cellular membranes controls Hedgehog signaling. *Nat. Chem. Biol.* **16**, 1303–1313 (2020).

15. A. F. Rudolf, M. Kinnebrew, C. Kowatsch, T Bertie Ansell, K. E. Omari, B. Bishop, E. Pardon, R. A. Schwab, T. Malinauskas, M. Qian, R. Duman, D. F. Covey, J. Steyaert, A. Wagner, M. S. P. Sansom, R. Rohatgi, C. Siebold, The morphogen Sonic hedgehog inhibits its receptor Patched by a pincer grasp mechanism. *Nat. Chem. Biol.* **15**, 975–982 (2019).

16. H. Nikaido, RND transporters in the living world. *Res. Microbiol.* **169**, 363–371 (2018).

17. S. W. Altmann, H. R. Davis Jr., L.-J. Zhu, X. Yao, L. M. Hoos, G. Tetzloff, S. P. N. Iyer, M. Maguire, A. Golovko, M. Zeng, L. Wang, N. Murgolo, M. P. Graziano, Niemann-Pick C1 like 1 protein is critical for intestinal cholesterol absorption. *Science* **303**, 1201–1204 (2004).

18. M. Kinnebrew, E. J. Iverson, B. B. Patel, G. V. Pusapati, J. H. Kong, K. A. Johnson, G. Luchetti, K. M. Eckert, J. G. Mc Donald, D. F. Covey, C. Siebold, A. Radhakrishnan, R. Rohatgi, Cholesterol accessibility at the ciliary membrane controls hedgehog signaling. *eLife* **8**, e50051 (2019).

19. J. Y. Song, A. M. Holtz, J. M. Pinskey, B. L. Allen, Distinct structural requirements for CDON and BOC in the promotion of Hedgehog signaling. *Dev. Biol.* **402**, 239–252 (2015).

20. R. Rohatgi, L. Milenkovic, M. P. Scott, Patched1 regulates Hedgehog signaling at the primary cilium. *Science* **317**, 372–376 (2007).

21. K. Petrov, B. M. Wierbowski, J. Liu, A. Salic, Distinct cation gradients power cholesterol transport at different key points in the Hedgehog signaling pathway. *Dev. Cell* **55**, 314–327.e7 (2020).

22. B. R. Myers, L. Neahring, Y. Zhang, K. J. Roberts, P. A. Beachy, Rapid, direct activity assays for Smoothened reveal Hedgehog pathway regulation by membrane cholesterol and extracellular sodium. *Proc. Natl. Acad. Sci.* **114**, E11141–E11150 (2017).

23. S. A. Hollingsworth, R. O. Dror, Molecular dynamics simulation for all. *Neuron* **99**, 1129–1143 (2018).

24. S. J. Marrink, V. Corradi, P. C. T. Souza, H. I. Ingólfsson, D. P. Tieleman, M. S. P. Sansom, Computational modeling of realistic cell membranes. *Chem. Rev.* **119**, 6184–6226 (2019).

25. G. Hedger, H. Koldsø, M. Chavent, C. Siebold, R. Rohatgi, M. S. P. Sansom, Cholesterol interaction sites on the transmembrane domain of the Hedgehog signal transducer and class F G protein-coupled receptor Smoothened. *Structure* **27**, 549–559.e2 (2019).

26. R. A. Corey, P. J. Stansfeld, M. Sansom, The energetics of protein-lipid interactions as viewed by molecular simulations. *Biochem. Soc. Trans.* **48**, 25–37 (2020).

27. G. Bussi, A. Laio, Using metadynamics to explore complex free-energy landscapes. *Nat. Rev. Phys.* **2**, 200–212 (2020).

28. A. S. J. S. Mey, B. K. Allen, H. E. Bruce Macdonald, J. D. Chodera, D. F. Hahn, M. Kuhn, J. Michel, D. L. Mobley, L. N. Naden, S. Prasad, A. Rizzi, J. Scheen, M. R. Shirts, G. Tresadern, H. Xu, Best practices for alchemical free energy calculations. *Living J. Comput. Mol. Sci.* **2**, 18378 (2020).

29. I. Deshpande, J. Liang, D. Hedeen, K. J. Roberts, Y. Zhang, B. Ha, N. R. Latorraca, B. Faust, R. O. Dror, P. A. Beachy, B. R. Myers, A. Manglik, Smoothened stimulation by membrane sterols drives Hedgehog pathway activity. *Nature* **571**, 284–288 (2019).

30. M. Kinnebrew, R. E. Woolley, T. B. Ansell, E. F. X. Byrne, S. Frigui, G. Luchetti, R. Sircar, S. Nachtergaele, L. Mydock-McGrane, K. Krishnan, S. Newstead, M. S. P. Sansom, D. F. Covey, C. Siebold, R. Rohatgi, Patched 1 regulates Smoothened by controlling sterol binding to its extracellular cysteine-rich domain. *Sci. Adv.* **8**, eabm5563 (2022).

31. C. Zhong, B. Wang, Regulation of cholesterol binding to the receptor Patched1 by its interactions with the ligand Sonic Hedgehog (Shh). *Front. Mol. Biosci.* **9**, 831891 (2022).

32. S. J. Marrink, H. J. Risselada, S. Yefimov, D. P. Tieleman, A. H. de Vries, The MARTINI force field: Coarse grained model for biomolecular simulations. *J. Phys. Chem. B* **111**, 7812–7824 (2007).

33. I. Ermilova, A. P. Lyubartsev, Cholesterol in phospholipid bilayers: Positions and orientations inside membranes with different unsaturation degrees. *Soft Matter* **15**, 78–93 (2019).

34. W. F. D. Bennett, D. P. Tieleman, Molecular simulation of rapid translocation of cholesterol, diacylglycerol, and ceramide in model raft and nonraft membranes. *J. Lipid Res.* **53**, 421–429 (2012).

35. M. Aldeghi, J. P. Bluck, P. C. Biggin, Absolute alchemical free energy calculations for ligand binding: A beginner’s guide. *Methods Mol. Biol.* **1762**, 199–232 (2018).

36. C.-C. Su, P. A. Klenotic, J. R. Bolla, G. E. Purdy, C. V. Robinson, E. W. Yu, MmpL3 is a lipid transporter that binds trehalose monomycolate and phosphatidylethanolamine. *Proc. Natl. Acad. Sci. U.S.A.* **166**, 11241–11246 (2019).

37. J. Taipale, M. K. Cooper, T. Maiti, P. A. Beachy, Patched acts catalytically to suppress the activity of Smoothened. *Nature* **418**, 892–896 (2002).

38. J. Briscoe, Y. Chen, T. M. Jessell, G. Struhl, A hedgehog-insensitive form of Patched provides evidence for direct long-range morphogen activity of Sonic hedgehog in the neural tube. *Mol. Cell* **7**, 1279–1291 (2001).

39. D. Du, X. Wang-Kan, A. Neuberger, H. W. van Veen, K. M. Pos, L. J. V. Piddock, B. F. Luisi, Multidrug efflux pumps: Structure, function and regulation. *Nat. Rev. Microbiol.* **16**, 523–539 (2018).

40. X. C. Zhang, M. Liu, L. Han, Energy coupling mechanisms of AcrB-like RND transporters. *Biophys. Rep.* **3**, 73–84 (2017).

41. M. Kinnebrew, G. Luchetti, R. Sircar, S. Frigui, L. V. Viti, T. Naito, F. Beckert, Y. Saheki, C. Siebold, A. Radhakrishnan, R. Rohatgi, Patched 1 reduces the accessibility of cholesterol in the outer leaflet of membranes. *eLife* **10**, e70504 (2021).

42. P. J. Stansfeld, J. E. Goose, M. Caffrey, E. P. Carpenter, J. L. Parker, S. Newstead, M. S. P. Sansom, MemProtMD: Automated insertion of membrane protein structures into explicit lipid membranes. *Structure* **23**, 1350–1361 (2015).

43. T. Eicher, M. A. Seeger, C. Anselmi, W. Zhou, L. Brandstätter, F. Verrey, K. Diederichs, J. D. Faraldo-Gómez, K. M. Pos, Coupling of remote alternating-access transport mechanisms for protons and substrates in the multidrug efflux pump AcrB. *eLife* **3**, e03145 (2014).

44. N. Fischer, C. Kandt, Three ways in, one way out: Water dynamics in the trans-membrane domains of the inner membrane translocase AcrB. *Proteins* **79**, 2871–2885 (2011).

45. R. Burke, D. Nellen, M. Bellotto, E. Hafen, K A Senti, B J Dickson, K. Basler, Dispatched, a novel sterol-sensing domain protein dedicated to the release of cholesterol-modified Hedgehog from signaling cells. *Cell* **99**, 803–815 (1999).

46. Q. Wang, D. E. Asarnow, K. Ding, R. K. Mann, J. Hatakeyama, Y. Zhang, Y. Ma, Y. Cheng, P. A. Beachy, Dispatched uses Na+ flux to power release of lipid-modified Hedgehog. *Nature* **599**, 320–324 (2021).

47. C. Wicking, S. Shanley, I. Smyth, S. Gillies, K. Negus, S. Graham, G. Suthers, N. Haites, M. Edwards, B. Wainwright, G. Chenevix-Trench, Most germ-line mutations in the nevoid basal cell carcinoma syndrome lead to a premature termination of the patched protein, and no genotype- phenotype correlations are evident. *Am. J. Hum. Genet.* **60**, 21–26 (1997).

48. E. C. Bailey, L. Milenkovic, M. P. Scott, J. F. Collawn, R. L. Johnson, Several PATCHED1 missense mutations display activity in patched1-deficient fibroblasts. *J. Biol. Chem.* **277**, 33632–33640 (2002).

49. H. Tukachinsky, K. Petrov, M. Watanabe, A. Salic, Mechanism of inhibition of the tumor suppressor Patched by Sonic Hedgehog. *Proc. Natl. Acad. Sci. U.S.A.* **113**, E5866–E5875 (2016).

50. L. E. Weiss, L. Milenkovic, J. Yoon, T. Stearns, W. E. Moerner, Motional dynamics of single Patched1 molecules in cilia are controlled by Hedgehog and cholesterol. *Proc. Natl. Acad. Sci. U.S.A.* **116**, 5550–5557 (2019).

51. X. Li, J. Wang, E. Coutavas, H. Shi, Q. Hao, G. Blobel, Structure of human Niemann-Pick C1 protein. *Proc. Natl. Acad. Sci. U.S.A.* **113**, 8212–8217 (2016).

52. X. Gong, H. Qian, X. Zhou, J. Wu, T. Wan, P. Cao, W. Huang, X. Zhao, X. Wang, P. Wang, Y. Shi, G. F. Gao, Q. Zhou, N. Yan, Structural insights into the Niemann-Pick C1 (NPC1)-mediated cholesterol transfer and ebola infection. *Cell* **165**, 1467–1478 (2016).

53. X. Gong, H. Qian, X. Zhou, J. Wu, T. Wan, P. Cao, W. Huang, X. Zhao, X. Wang, P. Wang, Y. Shi, G. F. Gao, Q. Zhou, N. Yan, 3.3 Å structure of Niemann–Pick C1 protein reveals insights into the function of the C-terminal luminal domain in cholesterol transport. *Proc. Natl. Acad. Sci. U.S.A.* **114**, 9116–9121 (2017).

54. T. Long, X. Qi, A. Hassan, Q. Liang, J. K. De Brabander, X. Li, Structural basis for itraconazole-mediated NPC1 inhibition. *Nat. Commun.* **11**, 152 (2020).

55. M. B. L. Winkler, R. T. Kidmose, M. Szomek, K. Thaysen, S. Rawson, S. P. Muench, D. Wüstner, B. P. Pedersen, Structural insight into eukaryotic sterol transport through Niemann-Pick type C proteins. *Cell* **179**, 485–497.e18 (2019).

56. R. E. Infante, M. L. Wang, A. Radhakrishnan, H. J. Kwon, M. S. Brown, J. L. Goldstein, NPC2 facilitates bidirectional transfer of cholesterol between NPC1 and lipid bilayers, a step in cholesterol egress from lysosomes. *Proc. Natl. Acad. Sci. U.S.A.* **105**, 15287–15292 (2008).

57. M. Delling, P. G. Decaen, J. F. Doerner, S. Febvay, D. E. Clapham, Primary cilia are specialized calcium signalling organelles. *Nature* **504**, 311–314 (2013).

58. T. L. Steck, J. Ye, Y. Lange, Probing red cell membrane cholesterol movement with cyclodextrin. *Biophys. J.* **83**, 2118–2125 (2002).

59. M. N. Melo, H. I. Ingólfsson, S. J. Marrink, Parameters for Martini sterols and hopanoids based on a virtual-site description. *J. Chem. Phys.* **143**, 243152 (2015).

60. R. A. Corey, O. N. Vickery, M. S. P. Sansom, P. J. Stansfeld, Insights into membrane protein–lipid interactions from free energy calculations. *J. Chem. Theory Comput.* **15**, 5727–5736 (2019).

61. M. J. Abraham, T. Murtola, R. Schulz, S. Páll, J. C. Smith, B. Hess, E. Lindahl, GROMACS: High performance molecular simulations through multi-level parallelism from laptops to supercomputers. *SoftwareX* **1-2**, 19–25 (2015).

62. J. S. Hub, B. L. De Groot, D. Van Der Spoel, g_wham—A free weighted histogram analysis implementation including robust error and autocorrelation estimates. *J. Chem. Theory Comput.* **6**, 3713–3720 (2010).

63. M. Souaille, B. Roux, Extension to the weighted histogram analysis method: Combining umbrella sampling with free energy calculations. *Comput. Phys. Commun.* **135**, 40–57 (2001).

64. R. Salari, T. Joseph, R. Lohia, J. Hénin, G. Brannigan, A streamlined, general approach for computing ligand binding free energies and its application to GPCR-bound cholesterol. *J. Chem. Theory Comput.* **14**, 6560–6573 (2018).

65. M. Parrinello, A. Rahman, Polymorphic transitions in single crystals: A new molecular dynamics method. *J. Appl. Phys.* **52**, 7182–7190 (1981).

66. M. Bonomi, D. Branduardi, G. Bussi, C. Camilloni, D. Provasi, P. Raiteri, D. Donadio, F. Marinelli, F. Pietrucci, R. A. Broglia, M. Parrinello, PLUMED: A portable plugin for free-energy calculations with molecular dynamics. *Comput. Phys. Commun.* **180**, 1961–1972 (2009).

67. G. A. Tribello, M. Bonomi, D. Branduardi, C. Camilloni, G. Bussi, PLUMED 2: New feathers for an old bird. *Comput. Phys. Commun.* **185**, 604–613 (2014).

68. M. R. Shirts, J. D. Chodera, Statistically optimal analysis of samples from multiple equilibrium states. *J. Chem. Phys.* **129**, 124105 (2008).

69. P. V. Klimovich, M. R. Shirts, D. L. Mobley, Guidelines for the analysis of free energy calculations. *J. Comput. Aided Mol. Des.* **29**, 397–411 (2015).

70. J. Huang, A. D. MacKerell Jr., CHARMM36 all-atom additive protein force field: Validation based on comparison to NMR data. *J. Comput. Chem.* **34**, 2135–2145 (2013).

71. S. Nosé, A molecular dynamics method for simulations in the canonical ensemble. *Mol. Phys.* **52**, 255–268 (1984).

72. W. G. Hoover, Canonical dynamics: Equilibrium phase-space distributions. *Phys. Rev. A* **31**, 1695–1697 (1985).

73. U. Essmann, L. Perera, M. L. Berkowitz, T. Darden, H. Lee, L. G. Pedersen, A smooth particle mesh Ewald method. *J. Chem. Phys.* **103**, 8577–8593 (1995).

74. B. Hess, H. Bekker, H. J. C. Berendsen, J. G. E. M. Fraaije, LINCS: A linear constraint solver for molecular simulations. *J. Comput. Chem.* **18**, 1463–1472 (1997).

75. J. Elegheert, E. Behiels, B. Bishop, S. Scott, R. E. Woolley, S. C. Griffiths, E. F. X. Byrne, V. T. Chang, D. I. Stuart, E. Y. Jones, C. Siebold, A. R. Aricescu, Lentiviral transduction of mammalian cells for fast, scalable and high-level production of soluble and membrane proteins. *Nat. Protoc.* **13**, 2991–3017 (2018).

76. D. H. de Jong, G. Singh, W. F. D. Bennett, C. Arnarez, T. A. Wassenaar, L. V. Schäfer, X. Periole, D. P. Tieleman, S. J. Marrink, Improved parameters for the martini coarse-grained protein force field. *J. Chem. Theory Comput.* **9**, 687–697 (2013).

77. X. Periole, M. Cavalli, S.-J. Marrink, M. A. Ceruso, Combining an elastic network with a coarse-grained molecular force field: Structure, dynamics, and intermolecular recognition. *J. Chem. Theory Comput.* **5**, 2531–2543 (2009).

78. T. A. Wassenaar, H. I. Ingólfsson, R. A. Böckmann, D. P. Tieleman, S. J. Marrink, Computational lipidomics with *insane* : A versatile tool for generating custom membranes for molecular simulations. *J. Chem. Theory Comput.* **11**, 2144–2155 (2015).

79. G. Bussi, D. Donadio, M. Parrinello, Canonical sampling through velocity rescaling. *J. Chem. Phys.* **126**, 014101 (2007).

80. W. Humphrey, A. Dalke, K. Schulten, VMD: Visual molecular dynamics. *J. Mol. Graph.* **14**, 33–38 (1996).

81. A. Fiser, A. Ŝali, Modeller: Generation and refinement of homology-based protein structure models. *Methods Enzymol.* **374**, 461–491 (2003).

82. J. C. Gordon, J. B. Myers, T. Folta, V. Shoja, L. S. Heath, A. Onufriev, H++: A server for estimating pKas and adding missing hydrogens to macromolecules. *Nucleic Acids Res.* **33**, 368–371 (2005).

83. C. R. Søndergaard, M. H. M. Olsson, M. Rostkowski, J. H. Jensen, Improved treatment of ligands and coupling effects in empirical calculation and rationalization of pKa values. *J. Chem. Theory Comput.* **7**, 2284–2295 (2011).

84. S. Jo, X. Cheng, S. M. Islam, L. Huang, H. Rui, A. Zhu, H. S. Lee, Y. Qi, W. Han, K. Vanommeslaeghe, A. D. MacKerell Jr., B. Roux, W. Im, CHARMM-GUI PDB manipulator for advanced modeling and simulations of proteins containing nonstandard residues. *Adv. Protein Chem. Struct. Biol.* **96**, 235–265 (2014).

85. S. Jo, J. B. Lim, J. B. Klauda, W. Im, CHARMM-GUI Membrane Builder for mixed bilayers and its application to yeast membranes. *Biophys. J.* **97**, 50–58 (2009).

86. CHARMM-GUI *Membrane Builder* for complex biological membrane simulations with glycolipids and lipoglycans. *J. Chem. Theory Comput.* **15**, 775–786 (2019).

87. W. L. Jorgensen, J. D. Madura, Temperature and size dependence for Monte Carlo simulations of TIP4P water. *Mol. Phys.* **56,** 1381–1392 (1985).

88. J. Gumbart, F. Khalili-Araghi, M. Sotomayor, B. Roux, Constant electric field simulations of the membrane potential illustrated with simple systems. *Biochim. Biophys. Acta Biomembr.* **1818**, 294–302 (2012).

89. W. Song, R. A. Corey, T. B. Ansell, C. K. Cassidy, M. R. Horrell, A. L. Duncan, P. J. Stansfeld, M. S. P. Sansom, PyLipID: A Python package for analysis of protein-lipid interactions from molecular dynamics simulations. *J. Chem. Theory Comput.* **18**, 1188–1201 (2022).
